# A comprehensive survey of the grapevine VQ gene family and its transcriptional correlation with WRKY proteins

**DOI:** 10.3389/fpls.2015.00417

**Published:** 2015-06-12

**Authors:** Min Wang, Alessandro Vannozzi, Gang Wang, Yan Zhong, Massimiliano Corso, Erika Cavallini, Zong-Ming (Max) Cheng

**Affiliations:** ^1^Fruit Crop Systems Biology Laboratory, College of Horticulture, Nanjing Agricultural UniversityNanjing, China; ^2^Institute of Botany, Jiangsu Province and the Chinese Academy of SciencesNanjing, China; ^3^Department of Agronomy, Food, Natural Resources, Animals and Environment, University of PadovaLegnaro, Italy; ^4^Department of Biotechnology, University of VeronaVerona, Italy

**Keywords:** grapevine, VQ gene family, gene expression, drought treatment, biotic stress, WRKY protein, co-expression network

## Abstract

WRKY proteins are a class of transcription factors (TFs) involved in the regulation of various physiological processes, including the plant response to biotic and abiotic stresses. Recent studies in *Arabidopsis* have revealed that some WRKY TFs interact with a class of proteins designed as VQ proteins because of their typical conserved motif (FxxxVQxLTG). So far, no information is available about the genomic organization and the function of VQ motif-containing protein in grapevine (*Vitis vinifera* L). In the current study, we analyzed the 12X V1 prediction of the nearly homozygous PN40024 genotype identifying up to 18 predicted VQ genes (*VvVQ*). *VvVQs* phylogenetic and bioinformatic analyses indicated that the intron-exon structures and motif distribution are highly divergent between different members of the grapevine VQ family. Moreover, the analysis of the *V. vinifera* cv. Corvina expression atlas revealed a tissue- and stage-specific expression of several members of the family which also showed a significant correlation with WRKY TFs. Grapevine *VQ* genes also exhibited altered expression in response to drought, powdery mildew infection, salicylic acid (SA) and ethylene (ETH) treatments. The present study represents the first characterization of *VQ* genes in a grapevine genotype and it is a pivotal foundation for further studies aimed at functionally characterizing this mostly unknown grapevine multigenic family.

## Introduction

WRKY proteins are a class of transcription factors (TFs) found almost exclusively in plants, which have been associated to the control of a broad range of biological processes including response to biotic and abiotic stresses (Journot-Catalino et al., 2006; Liu et al., 2006; Xu et al., 2006; Zheng et al., 2006; Kim et al., 2008; Lai et al., 2008; Li et al., 2009), hormone signaling (Chen et al., 2010; Shang et al., 2010), secondary metabolism (Wang et al., 2010b; Suttipanta et al., 2011), and developmental processes (Johnson et al., 2002; Robatzek and Somssich, 2002; Miao et al., 2004; Luo et al., 2005; Jiang and Yu, 2009). In higher plants, genes encoding WRKY TFs are organized in large superfamilies as confirmed by recent genome-wide studies in species such as *Arabidopsis* (*Arabidopsis thaliana*; 74 genes), rice (*Oryza sativa;* 102 genes), and grapevine (*Vitis vinifera;* 59 genes) (Eulgem et al., 2000; Ramamoorthy et al., 2008; Wang et al., 2014).

One of the common defining features of WRKY TFs is their highly conserved DNA binding region, also called the WRKY domain, which consists of about 60 amino acids at the N-terminus, adjacent to a zinc-finger motif at the C-terminus. The conservation of the WRKY domain is mirrored by a remarkable conservation of its cognate binding site, the W-box (TTGACC/T), which is a common feature in all promoters of WRKY-regulated genes (Rushton et al., 1996; Eulgem et al., 2000).

It has been previously shown that interacting-partners such as co-activators, chromatin re-modelers, and enzymes modifying histones could modulate differential DNA-binding and transcription-regulatory activities of many WRKY TFs. Among these interacting partners is a class of proteins characterized by a short conserved VQ (FxxxVQxLTG) motif and generally designed as VQ proteins. The first VQ protein was identified in *Arabidopsis* by using a yeast two-hybrid assay with a MAP kinase, MPK4, as bait. The MPK4-interacting protein, designed as MPK Substrate (MKS) was found to be a 222-amino-acid protein member of a plant specific family including proteins from dicots and monocots sharing a VQ motif of unknown function (Andreasson et al., 2005). The VQ protein MKS forms complexes with AtWRKY25 and AtWRKY33, two group I WRKY TFs, involved in the regulation of plant defense responses (Andreasson et al., 2005). In absence of pathogen infection, MPK4 exists in nuclear complexes with WRKY33 through mutual interactions with MKS1 (Andreasson et al., 2005; Qiu et al., 2008). Upon *Pseudomonas* infection or flagellin treatment, activated MPK4 phosphorylates MKS1 and releases WRKY33, which then targets the expression of PAD3, a gene encoding for a protein involved in the biosynthesis of the phytoalexin camalexin (Andreasson et al., 2005). Subsequently, Lai et al. (2011) identified and functionally characterized other two VQ proteins, namely SIGMA FACTOR BINDING PROTEIN 1 (SIB1) and SIB2, which positively interact with WRKY33 in the plant defense against necrotrophic pathogens. The interaction between VQs and WRKY TFs was also described in the plant response to abiotic environmental stresses. In fact, a study aimed at clarifying the role of WRKY8 in salt tolerance in *Arabidopsis* showed that the interaction of this TF with the protein VQ9 results in the decrease of the WRKY8-DNA-binding activity and in negative regulation of salinity stress responses (Hu et al., 2013). Finally, another *Arabidopsis* VQ domain-containing protein, known as HAIKU1 (IKU1, called AtVQ14) was demonstrated to interact with WRKY10 to regulate endosperm growth and seed size (Wang et al., 2010a).

A genome wide screening of the *VQ* genes in *Arabidopsis* led to the identification of 34 members. These proteins were found to interact exclusively with group I and II-c WRKY TFs and their overexpression led to altered flowering and growth phenotypes, which supports the hypothesis that this gene family could be involved not only in the plant responses to stresses, but, as observed for WRKYs, also in developmental processes (Cheng et al., 2012). The conserved valine and glutamine residues in the short VQ motif are important for the physical interaction with WRKY C-terminal domains. Recently, a comprehensive analysis of VQ motif containing genes in rice led to the identification of 39 VQ members. These genes, similarly to what observed in *Arabidopsis*, were shown to be involved in disease resistance and in the plant response to environmental stresses (Kim et al., 2013).

Cultivated grapevine (*V. vinifera* L.) is an economically important fruit crop. A first genome sequence assembly of a highly homozygous line of *V. vinifera* Pinot Noir (PN40024) was published in 2007 (Jaillon et al., 2007), followed by a second assembly obtained from a highly heterozygous Pinot Noir line (PN ENTAV 115) (Velasco et al., 2007). These assemblies have been uploaded and improved by the grapevine community during the last few years and represent an invaluable resource for many studies. So far, many gene families and superfamilies have been identified and characterized in grapevine, however, there has been no systematic analysis of *VQ* genes family. In the current study, a genomic characterization of the VQ family in the 12X V1 prediction of the near homozygous PN40024 genotype led to the identification of 18 predicted *VvVQ* genes. The genomic sequences and phylogeny of all the *VvVQ* genes were analyzed, together with their expression profile at different developmental stages and in response to different biotic and abiotic stresses. Moreover, based on coexpression analyses we raised some hypotheses on putative interactions between VQ motif-containing proteins and several WRKY TFs, which has to be confirmed by functional studies. These analyses provide important information about the evolution and function of the grapevine *VQ* genes and represent the first pivotal foundation for further studies on the role of these genes in the regulation of developmental and defense mechanisms.

## Materials and methods

### Identification of grapevine VQ gene family members

The 12X V1 release of the *V. vinifera* PN40024 genome containing the most recently uploaded version of gene and protein predictions was retrieved from the CRIBI Biotechnology Center website (http://genomes.cribi.unipd.it/). *Arabidopsis* and rice genome sequences were obtained from the Arabidopsis Information Resource (TAIR; http://www.arabidopsis.org) and the Rice Genome Annotation project sites (http://rice.plantbiology.msu.edu/) respectively. The Hidden Markov Model (HMM) of the VQ motif (PF05678) was downloaded from the Pfam database (http://pfam.sanger.ac.uk/). A search of all grapevine VQ protein sequences was carried out with the HMM search tool (HMMER 3.0; http://hmmer.janelia.org/) using the VQ motif HMM profile as a query and default parameters (*E* < 0.01). All candidate grapevine VQ genes with non-redundant hits were retained and confirmed using the SMART software program (http://smart.embl-heidelberg.de/). Moreover, as a cross check, a Pfam-code protein domain search was performed directly using the query system interface of the CRIBI website (http://genomes.cribi.unipd.it/). Recently, a new prediction (V2) of the PN40024 12X coverage was released (Vitulo et al., 2014); sequences retrieved from the V1 were checked against the V2 in order to identify variants or discrepancies. Length of sequences, isoelectric points, and molecular weights of deduced polypeptides were calculated by the ExPasy website (http://web.expasy.org/protparam/). The protein subcellular location was predicted by the Wolf PSORT program (http://www.genscript.com/psort/wolf_psort.html).

### Sequence alignment and phylogenetic analysis

Multiple alignments of grapevine, *Arabidopsis* and rice VQ domain sequences were performed using MUSCLE with default options (Edgar, 2004). The phylogenetic tree was inferred based on the neighbor-joining (NJ) method with a Kimura 2-parameter model using MEGA 5.0 (Tamura et al., 2011). Bootstrap values were calculated for 1000 iterations. Moreover, an additional phylogenetic tree was constructed based on a MCMC (Bayesian Markov chain Monte Carlo) method with Poisson rate matrix amino-acid model by mean of MrBayes software (Huelsenbeck and Ronquist, 2001).

### Motif search and exon-intron structure analysis

The predicted *VQ* coding (CDS) and genomic sequences identified in the V1 prediction of the PN40024 genotype were used to infer the intron/exon structure using the online Gene Structure Display Server (GSDS: http://gsds.cbi.pku.edu.ch). To assess the structural divergence of grapevine *VQ* genes, the Multiple Expectation Maximization for Motif Elicitation (MEME) online program (http://meme.sdsc.edu/meme/itro.html) (Bailey et al., 2009) was used to identify motifs in the 18 identified grapevine VQ protein sequences. The optimized parameters of MEME used were the following: maximum number of motifs, 20; minimum motif width, 6; and maximum motif width, 50.

### Chromosomal locations and gene duplication

The chromosomal location of grapevine *VQ* genes was determined based on the 12X V1 release in the grapevine genome browser at the CRIBI website (http://genomes.cribi.unipd.it/). The distribution of *VvVQ* genes was drawn using MapInspect software (http://www.plantbreeding.wur.nl/UK/software_mapinspect/). The physical location of *Arabidopsis VQ* genes was mapped using the Chromosome Map Tool at TAIR (http://arabidopsis.org/jsp/ChromosomeMap/tool.jsp). *VQ* genes duplication events in *Arabidopsis* and grapevine were also investigated. Tandem duplicated genes defined as an array of two or more genes that were in the same phylogenetic group and found within a 100 kb genomic window (Yang et al., 2008). The information for segmental duplication was obtained from the Plant Genome Duplication Database (http://chibba.agtec.uga.edu/duplication/). Synteny blocks within the *Arabidopsis* genome and between grapevine and *Arabidopsis* genomes were downloaded from the Plant Genome Duplication Database and those containing grapevine *VQ* genes were identified.

### Promoter analysis

Sequences of 2000 bp upstream the coding regionof each predicted *VvVQ* gene were downloaded from the grapevine genome website CRIBI (http://genomes.cribi.unipd.it/) and screened for *cis*-acting elements using PlantCARE (http://bioinformatics.psb.ugent.be/webtools/plantcare/html/) according to Liu et al. (2009). PlantCARE software was also used for identifying putative plant-specific TF binding sites (TFBSs) in a given DNA sequence.

### Public microarray-based expression analysis of *VvVQ* genes

The expression patterns of *VvVQ* genes, predicted from an analysis of the grapevine genome, was determined in a global *V. vinifera* cv. Corvina (clone 48) gene expression atlas of different organs at various developmental stages. Microarray data were downloaded from the Gene Expression Omnibus under the series entry GSE36128 (http://www.ncbi.nlm.nih.gov/geo/) (Fasoli et al., 2012) and includes the following tissues: *in vitro* roots, green stem, buds after budburst (rosette of leaf tips visible), young leaves (leaves collected from shoots with only five leaves), senescing leaves (leaves at the beginning of leaf-fall), berry rachis (from fruit-set to ripening), flowers (50% cap-fall), and berry pericarp (from fruit set to ripe). In addition, berries were also examined which had undergone post-harvesting withering for 1–3 months after harvest. Data were analyzed and graphically represented using MeV (Multi Experiment Viewer) software (Saeed et al., 2006). Data obtained from the *V. vinifera* cv. Corvina expression atlas were normalized based on the mean expression value of each gene in all tissues/organs analyzed.

### Plant growth and stress treatments

Pinot noir 40024 *in vitro*-grown plants were grown at 25°C under long-day conditions (16 h/8 h light/dark) in a culture room. For the drought treatment, 5-week-old *in vitro* PN40024 plants were transplanted into pots and acclimated in an environmental chamber at 23 ± 2°C, a 16 h/8 h light/dark photoperiod [100 μmol/(m^2^• s)], and with a relative humidity of 70–80%. Potted plants were first kept in well-watered conditions until the plants were approximately 40 cm tall with 14 leaves. One group of plants was maintained under well-watered conditions as control, while the other three groups of plants received no water (drought treatment). Samples were harvested at 0, 4, 8, and 12 days after water was withheld and immediately frozen in liquid nitrogen and stored at −70°C until further analysis. Each time point had a corresponding control. For each treatment, leaves from three independent plants, considered as three biological replicates were sampled. For the biotic stress treatment, a local strain of *Erysiphe necator* was isolated from infected leaves collected from the “Meiguixiang” (Muscat) variety belonging to the Nanjing Agricultural University grape germoplasm resources and maintained on leaves of *V. vinifera* Pinot noir PN40024 glasshouse-grown plants. Inoculation of grapevine PN40024 leaves were performed by using the compression method pressing infected-leaves on healthy leaves of similar size. PN40024 leaves were collected 0, 4, 12, and 24 h after inoculation and immediately frozen in liquid nitrogen and stored at −70°C until subsequent analysis could take place. For a mock control, similar sized, Pinot noir healthy leaves were pressed onto *in vitro* leaves of PN40024. For hormone treatments, 5-week old *in vitro* grapevine plants were sprayed with 5 mM SA or 0.5 g/L ethephon. Plants sprayed with sterile distilled water were collected as control. Samples were harvested at 0, 4, 12, and 24 h after treatment. In these experiments, each sample (treated and untreated) was collected in three biological replicates.

### Quantitative RT-PCR expression analysis of grapevine *VvVQ* genes

Quantitative RT-PCR was performed on an ABI 7300 Real Time PCR System (Applied Biosystems, USA) using the SYBR-Green (Takara, Japan) method. Designing of the oligonucleotide primers was based on the 3'-untranslated region and the 3'terminal sequences of the predicted coding region of each gene using Beacon designer software (version 2) and are listed in Table S1. Primers were checked using the BLAST tool at NCBI and the dissociation curve for each primer set was analyzed after the PCR reaction to confirm specificity. The specificity of PCR products was also verified by cloning the amplicons into the pMD19-T Vector (Takara). Cloned products were subsequently sequenced and aligned to the grapevine reference genome. Each reaction was carried out in a volume of 20 μl, containing 10 μl SYBR, 8.6 μl ddH_2_O, 1 μl diluted template (1 μl of the generated first-strand cDNA diluted by 9 μl ddH_2_O), and 0.2 μl of each gene-specific primers. The following PCR program was used: 94°C for 30 s (pre-denaturation) followed by 40 cycles at 94°C for 30 s (denaturation), 60°C for 20 s (primer annealing) and 72°C for 43 s (extension and obtaining the fluorescent signal). The specificity of the reactions was verified by melting curve analysis. The baseline and threshold cycles (Ct) were automatically determined by the ABI software. Three technical replicates were used for each biological replicate. The actin-101-like gene (*VIT_12S0178g00200*) whose expression levels remained nearly constant under all experimental conditions (CT was approximately 21 in all samples considered) was used as an internal control. The relative expression for all selected genes was calculated using the formula the 2^−ΔΔCt^ method, where ΔΔCt= (Ct _target gene_ − Ct _actin_) _treatment_ − (Ct _target gene_ − Ct _actin_)_ck_(Yuan et al., 2006). To visualize the relative fold difference, all data were represented by setting the relative expression level of 0-point treatment as 1, “above 1” and “below 1” are considered as up or down regulation during the stress treatment.

### Statistical and correlation network analyses

Statistic analyses were performed using Dunnett's two-tailed *t*-test. Mean values ± SD of at three replicates were presented, and significant differences relative to controls were given at ^*^P ≤ 0.05 and ^**^P ≤ 0.01. Pearson correlation (r) and respective FDR adjusted *p*-values were calculated between expression values of *WRKY* genes belonging to group I and II-c and all VQs, by using R programming language (http://cran.r-project.org/). The correlation network analysis (http://www.cytoscape.org/) (Shannon et al., 2003) was performed with VQ and WRKY co-expressed genes that highlight a *r* > 0.6 and a FDR < 0.05.

## Results and discussion

### Identification and annotation of *VVvQ* genes

A search for genes encoding VQ proteins in the 12X V1 prediction of the PN40024 grapevine genome (http://genomes.cribi.unipd.it/) was performed, by using the HMM profile of the VQ motif (PF05678) as query. A total of 18 genes putatively encoding VQ proteins were identified in the grapevine reference genome and confirmed by a cross-check using the CRIBI interface for searching prediction based on Pfam codes. 16 *VQ* genes were located on 10 out of 19 grapevine chromosomes and named from *VvVQ1* to *VvVQ16* Figure S1). Two additional predicted genes (VIT_00s0304g00030 and VIT_00s0471g00020) were located within scaffolds not anchored to any chromosome (unknown chromosome, chrUn) and were arbitrarilly designated as *VvVQ17* and *VvVQ18*, respectively Figure S1). The compositional characteristics of the grapevine *VQ* genes, including the deduced protein length (aa), weight (KDa), and isoelectric point (pI) are listed in Table 1. Data indicated that the predicted *VvVQ* genes encode peptides ranging from 110 (VvVQ12) to 490 (VvVQ17) aa in length. As observed in *Arabidopsis*, the majority of members encoded small proteins with fewer than 300 aa residues (Cheng et al., 2012). The isoelectric point (pI) of the VQ proteins ranged from 4.42 (VvVQ9) to 10.95 (VvVQ3). Compared to *Arabidopsis* and rice (39 and 34 *VQ* genes, respectively) (Cheng et al., 2012; Kim et al., 2013), grapevine has the lowest number of members, even though the size of its genome (475 Mb) is larger than the *Arabidopsis* (125 Mb) and rice (389 Mb) ones. A possible explanation of the small size of the *VQ* family in grapevine is the fact that whereas two rounds of genome duplication (α and β) were described in *Arabidopsis* (Bowers et al., 2003), there was no genome-wide duplication in *Vitis* after a shared ancient γ triplication (Jaillon et al., 2007; Tang et al., 2008a,b). Moreover, it must be noted that this study was performed on the 12X V1 prediction, which is based on *in silico* analyses. We cannot rule out the possibility that additional VQ were missed because of gaps in the genome assembly or incomplete/wrong gene predictions.

**Table 1:** **Properties of grapevine VQ proteins**.

**Proposed name**	**PN4002412X V1 ID**	**PN4002412X V2 ID**	**ORF (aa)**	**pI**	**MW (kDa)**	**Comments**
VvVQ1	VIT_01s0011g01350	VIT_201s0011g01350.1	195	6.83	21445.7	nucl
VvVQ2	VIT_01s0011g03650	VIT_201s0011g03650.1	196	6.64	21395.7	nucl
VvVQ3	VIT_02s0012g01280	VIT_202s0012g01280.1	286	10.95	31050.6	nucl
VvVQ4	VIT_04s0008g06930	VIT_204s0008g06930.1	183	10.17	19886.4	cyto
VvVQ5	VIT_08s0040g00540	VIT_208s0040g00540.1	219	7.85	23787.8	nucl
VvVQ6	VIT_08s0007g05180	VIT_208s0007g05180.1	143	4.67	16007.0	cyto
VvVQ7	VIT_08s0007g06470	VIT_208s0007g06470.1	157	8.47	17361.9	cyto
VvVQ8	VIT_09s0002g07540	VIT_209s0002g07540.1	186	9.96	20068.6	chlo
VvVQ9	VIT_12s0028g00500	VIT_212s0028g00500.1	183	4.42	19934.5	chlo
VvVQ10	VIT_13s0084g00670	VIT_213s0084g00670.1	124	7.85	14009.0	chlo
VvVQ11	VIT_13s0156g00160	VIT_213s0156g00160.1	160	9.22	18343.1	nucl
VvVQ12	VIT_14s0081g00190	VIT_214s0081g00190.1	110	7.81	11938.3	nucl
VvVQ13	VIT_18s0001g05400	VIT_218s0001g05400.1	464	6.63	48960.6	nucl
VvVQ14	VIT_18s0001g06350	VIT_218s0001g06350.1	335	10.63	36615.3	nucl
VvVQ15	VIT_18s0166g00090	VIT_218s0166g00090.1	252	7.96	27075.6	nucl
VvVQ16	VIT_19s0014g02490	VIT_219s0014g02490.1	210	9.60	22922.8	nucl
VvVQ17	VIT_00s0304g00030	–	490	7.89	52552.9	nucl
VvVQ17	–	VIT_200s0304g00030.1	460	6.65	49000.8	nucl
VvVQ18	VIT_00s0471g00020	VIT_200s0471g00020.1	187	9.30	20343.1	chlo

Based on Wang et al. (2014), also the number of grapevine *WRKYs* is lower when compared with *Arabidopsis* and rice. Similar sizes of the group I and IIc *WRKYs* and *VQ* multigenic families were reported in *Arabidopsis* (Cheng et al., 2012), but despite that, interaction partnership between the WRKY and VQ proteins was found not to be highly specific. In fact, yeast two-hybrid assays showed that a single VQ protein is able to interact with multiple WRKYs, and different VQ proteins have partially overlapping pools of interacting WRKY partners. Thus, there might not be a biunivocal relation between a given WRKY TF and a VQ protein.

### Multiple sequence alignment and structural analysis

**Figure 1:**
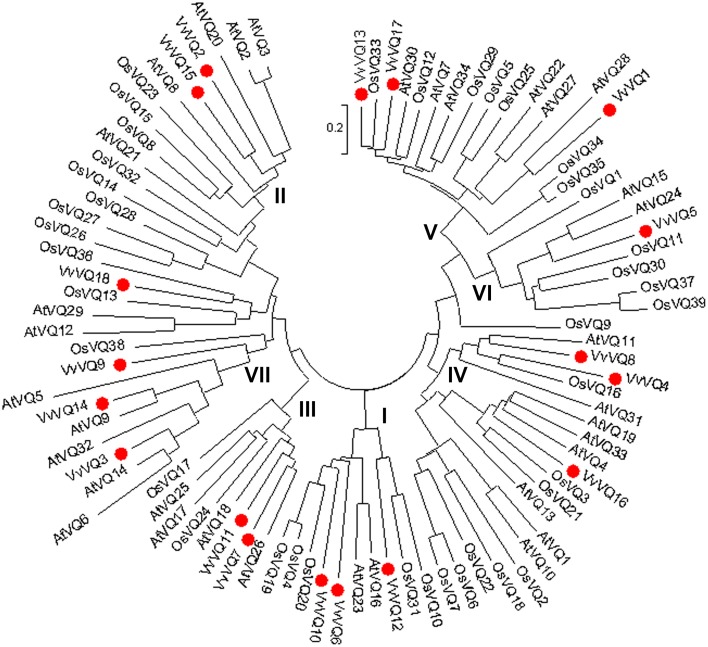
**Phylogenetic analysis of *Arabidopsis*, rice, and grapevine VQ proteins**. The unrooted tree, based on the core VQ domains in the three species, was constructed using the Neighbor-Joining (NJ) method (bootstrap 1000 replicates) using MEGA5 software.

**Figure 2:**
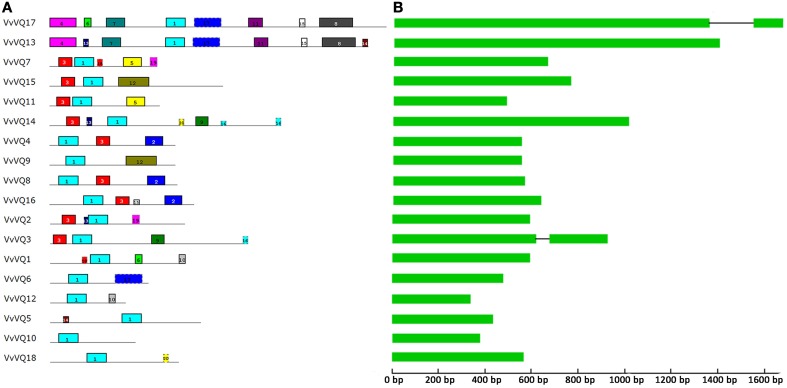
**Structural analysis of grapevine *VQ* genes**. **(A)** The motif composition within each VvVQ protein. The motifs, numbered 1–20, are displayed in different colored boxes. The sequence information for each motif is provided in Table 2. **(B)** Exon/intron structures of grapevine *VQ* genes. Exons were drawn to scale and are representred by boxes. Solid lines connecting two exons represent introns.

The deduced VQ domains of the 18 VvVQ proteins identified in the grapevine reference genome span across 57 aa in length and were aligned by means of MUSCLE software as reported in Figure S2. The core amino acid sequence, which was found to be conserved within all grapevine VQ proteins, is FXXXVQX(L/V/F)TG. No alternative variants, such as the VH core sequence in rice (Kim et al., 2013) were found in grapevine. An unrooted tree was constructed with the 18, 39, and 34 VQ-domainsequences found in grapevine, rice, and *Arabidopsis*, respectively, using the NJ method (Figure 1). Based on the rice VQ domain classification and the phylogenetic tree (Kim et al., 2013), the grapevine VQ domains were classified into seven subgroups even though this subdivision has to be considered with the due prudence considering the low bootstrap values obtained likely due to the divergent sequences of the protein family as proposed by Cheng et al. (2012) analysing the VQ family in *Arabidopsis*. It is not surprising that the phylogenetic tree used produced with a different Bayesian-based alghoritm (Figure S3) gave a different clusterization resect to what obtained with the NJ method in this analysis and in the one performed by Kim et al. (2013). As shown in Figure 1, only one VvVQ protein was located in group VI. In group II, V, and VI, the VQ domains of the genes in the grapevine and *Arabidosis* clustered together, while the genes of the rice VQ domains clustered by themselves. This further confirmed that grapevine has a close evolutionary relationship with *Arabidopsis*. Figure 2 illustrated the pattern of motifs detected within each protein, and the exon-intron structure of the grapevine *VQ* genes. A total number of 20 different motifs, ranging from 6 to 50 amino acids in length, were detected using MEME 4.3.0 software (http://meme.sdsc.edu/meme/intro.html) (Bailey et al., 2009) (Figure 2A, Table 2). As illustrated in Figure 2A, motif 1 is the VQ motif widely distributed in all 18 proteins. The differences in the type and number of motifs within the VvVQ proteins represent the structural basis for the diversity in gene function.

**Table 2:** **Multilevel Consensus Sequence for each motif of VvVQ proteins as predicted by MEME program**.

**Motif**	***E*-value**	**Sequence**
motif1	2.9e−128	[RV][AI][SI]YRS[PS][PT][TK][VIF][YVI][TQK][TA]DASNF[RMK] [AD][LV]VQ[ERQ]LTG
motif2	1.6e−012	EE[RK]AIAEKGFY[LF][HL]PSP[ML]STPR[TGD]SE
motif3	1.2e−006	GPR[PR]SP[LF]K[LM][NH][KE][DR][SR]H[KST]I[RS]K
motif4	2.8e−003	MDSGNS[GS]SMQSSSGGDEEY[DE]SR[AP]ESI [PS]AFLNP[PS]GH[FV]G
motif5	5.1e−003	E[KH][MI]W[RG][GD]ENS[NG]GF[LF][SG]GF[GA]DLDGFMQE
motif6	6.8e−003	HHH[SH]HHH[TR]H
motif7	1.3e−001	N[PA]NSLLNLD[TM]VWS[RK][TG]LRS[ED]PNCT[ED][IF]G
motif8	1.3e−001	[SC]W[RN]DG[LA]GS[SN][GE]G[SN][RH][GE]QL[RG]P[LF] NGNY[NG][ND]S[QP][RQ]V[TS][SN][GF]K[ML]N[YC]SASSS [DA]FH[PG][ED]K
motif9	2.6e+000	[WV][TH][HA][VA]AESPISAYMR[YL][LF]Q
motif10	4.6e+001	M[NL]D[QI]G[HF][IF]W
motif11	1.7e+002	YQLPSD[LP]G[FL][PV]KQPQN[LV]L[GN]M
motif12	2.3e+002	[VI]E[SK][TR][QK]V[SE]A[EC][ED]E[TK][KI][LK][KD]M[TQ][SA] [SL][GD]D[VF][PD][VM][VN][QE][GD][IA][GE] [VI][LG]G[GF][VQ][ED][RM][SE][SG]W[LF][PL][QG]I
motif13	6.9e+002	H[HQ]Q[YRN]H[PH]
motif14	5.2e+002	[VA]DSWI[SC]
motif15	7.5e+002	HGHVN[TA][NH]
motif16	1.0e+003	PRWRD[PI]
motif17	1.1e+003	R[LG]D[LE][FP][GP][TQA][MGA]S[TSD][IM][RPK][SPM][GE] [HQ]L[DE][PHD][VLA][PGF][PT][SPL][YQ][LP]L[RE] [PN][FS][TPA][QHG][KL][LV]Q[PS][TPN][LP][FW][YVA]
motif18	1.3e+003	KGC[RI][SK]K
motif19	7.1e+002	H[MC][HG]G[FA]G[ED]AQ
motif20	2.1e+003	[YV]P[WG]C[TS]F

Sequence comparisons of grapevine full-length cDNAs and genomic DNA revealed the number and position of exons and introns for each of the individual *VQ* genes. With the exception of two *VvVQ* genes (*VvVQ3* and *VvVQ17*), the majority of *VQ* genes in grapevine lack introns (Figure 2B), which is consistent with *Arabidopsis* and rice. A screening of the V2 prediction of the PN40024 genome, recently released by CRIBI Biotechnology Center (Vitulo et al., 2014) (http://genomes.cribi.unipd.it/grape/) indicates some discrepancies when compared with the V1 prediction. In fact, *VvVQ17*, which is predicted to contain an intron in the V1 release, does not appear to contain any intron in the V2. A comparison with the corresponding sequence in the PN ENTAV 115 genome (Velasco et al., 2007) confirmed that the right prediction is the most recent one (V2). Thus, among the 18 *VvVQ* genes, *VvVQ3* remains the only one presenting an intronic fragment in its gene structure. Although, the analysis of the exon-intron structure of VQ genes was performed on both the V1 and V2 version of the PN40024 genome and checked also in the PN ENTAV one, it is worth to point out that the analysis of gene structure was based on *in silico* predictions, and thus could carry imprecisions and errors before a good quality and reliable gene annotation will be improved in grapevine.

### Chromosomal locations and gene duplication

The chromosomal position of the 18 grapevine *VvVQ* genes and of 34 *AtVQ* genes was depicted in Figures S1, S4, respectively. The distribution of VQ genes among the chromosomes in the two species appeared to be different. As shown in Figure S4, *Arabidopsis* chromosome 1 (chr1) contained the highest density (32.35% of *VQ* genes) whereas chr4 and chr5 contained the lowest density (11.76% each) of *VQ* genes. Based on the plant genome duplication database, there were 10 segmental duplication events between chromosomes in *Arabidopsis* (Figure S4). In addition to these segmental duplications, it appeared that chr1 had undergone five tandem-duplication events (Yang et al., 2008). In relation to *VQ* gene expansion in *Arabidopsis*, segmental duplication was predominant, although tandem duplication was also involved. As previously indicated, 16 *VvVQ* genes were mapped to 10 different grapevine chromosomes, whereas two of them (*VvVQ17* and *VvVQ18*) were situated on unanchored contigs (chromosome unknown). Most of the grapevine chromosomes had only one single *VQ* member, although the number varied from 1 to 3. Unlike rice and *Arabidopsis*, no gene duplication events were observed in the *VQ* genes of grapevine. Syntenic regions between grapevine and *Arabidopsis* genomes were downloaded from the Plant Genome Duplication Database (http://chibba.agtec.uga.edu/duplication/) and syntenic blocks containing 4 *VQ* genes in grapevine and 5 *VQ* genes in *Arabidopsis* were identified (Table S2 and Figure S5). Three pairs of *VQ* genes (*VvVQ3*-*AtVQ32*, *VvVQ11*-*AtVQ15*, and *VvVQ15*-*AtVQ3*) between grapevine and *Arabidopsis* exhibited *VQ* gene correspondences. Each pair of genes clustered within the same clade in the phylogenetic tree, indicating that these genes were derived from a common ancestor. There was also one case (*VvVQ6*-*AtVQ17/AtVQ25*) where a single grapevine gene corresponded to multiple *Arabidopsis VQ* genes. Genomic comparisons represent a quick way to transfer knowledge acquired in one taxon to other taxa (Lyons et al., 2008). Thus, the abundance of information about gene function in *Arabidopsis* allows one to infer a potential gene function for orthologous genes in the syntenic region of other species, such as grapevine species.

### Identification of *cis*-elements in the promoter regions of *VvVQ* genes

In order to obtain useful information related to the regulatory mechanism of *VvVQ* gene expression, the putative transcription factor binding sites (TFBSs) in the 2000bp DNA sequences upstream the start codon of *VvVQ* genes were identified using PlantCARE (http://bioinformatics.psb.ugent.be/webtools/plantcare/html/) online software. Four categories of *cis*-acting elements were found to be the most represented in the promoter region of *VvVQ* genes in addition to the basic TATA and CAAT boxes (Table S3). The first class was constituted by “Light responsive elements,” such as the G-box (Menkens et al., 1995), I-box (Manzara et al., 1991), Box I, Sp1, and Box 4 (Lois et al., 1989), detected in most of *VvVQ* promoters. The second category of *cis*-acting element in the *VvVQ* promoter regions was constituted by hormone-responsive elements, such as ABRE (Simpson et al., 2003), P-box (Kim et al., 1992), and the TCA-element (Pastuglia et al., 1997). The CGTCA-motif (present in 77.8%, 14 out of 18 promoter sequences) and TGACG-motif (present in 83.3%, 15 out of 18 promoter sequences) are both *cis*-acting regulatory elements involved in methyl jasmonate (MeJA)-responsiveness (Rouster et al., 1997). The TCA-element (present in 77.8% of members, 14 out of 18 promoter sequences) is a *cis*-acting element involved in salicylic acid (SA) responsiveness. Both MeJA and SA are thought to play key roles in plant defense signaling (Gaffney et al., 1993; Xu et al., 1994). Therefore, it appears that most of the *VvVQ* genes may be involved in pathogen resistance. The ABRE element (72.2%, 13 of 18 promoter sequences) also accounts for a large proportion of hormone-related *cis*-acting elements in grapevine, suggesting that abscisic acid (ABA) may also regulate the expression of some *VvVQ* genes. The third major class of *cis*-acting elements consisted of TFBS related to external environmental stresses. 15 out of 18 *VvVQ* promoter sequences were found to contain a heat shock element HSE (83.3%) (Freitas and Bertolini, 2004), MBS, a *cis* element involved in drought response was detected in 10 out of 18 promoter sequences (55.6%) (Yamaguchi-Shinozaki and Shinozaki, 1993), and Box-W1, a fungal-elicitor-responsive element was also represented in 83.3% of sequences (15 out of 18 promoter sequences) (Rushton et al., 1996). The fourth most abundant class of *cis*-acting elements consisted of elements involved in various aspects of plant development. The Skn-1 motif which is involved in endosperm expression (Washida et al., 1999) is present in all *VvVQ* genes, with the exception of *VvVQ13* and *VvVQ17*. The mean number of copies (2,72) of this *cis*-acting element in the *VvVQ* gene family was higher than that of other types of *cis*-acting elements except for the TATA-box, CAAT-box, 5'UTR Py-rich stretch (Daraselia et al., 1996) and the unnamed-4, which indicate that most *VvVQ* genes could be involved in the process of endosperm development. The diversity in the types of *cis*-acting elements found in the upstream region of *VvVQ* genes indicates that these genes may have a wide range of functional roles, even thoughit is worth noting that the low number of plant *cis*-element currently annotated on databases represents a limitation in interpreting genomic data.

VQ proteins are likely to play an important role in plant growth, development, and response to environmental conditions acting as cofactors of group I and IIc WRKY TFs, as already proposed in *Arabidopsis* (Cheng et al., 2012). The average frequency of the W-boxes (TTGAC, WRKY-binding sites) in the 1.5-kb promoters regions of the 34 *Arabidopsis VQ* genes is approximately 3.8, which is substantially higher than the statistically expected frequencies (Dong et al., 2003). This suggests a complex regulatory mechanism in which the WRKY TFs regulate the expression of the *VQ* genes, that are in turn necessary as cofactors of the WRKY proteins (Cheng et al., 2012). In our analysis, W-box sequenceswere found in 14 out of 18 *VvVQ* genes, but the average frequency of W-boxes in the promoter of grapevine VQ genes was only 1.2. Based on a recent study (Corso, personal communication), the average frequency of TTGAC W-box in 10000 randomly chosen 2 Kb promoter regions is approximately 1.9. This comparison suggests a lower level of transcriptional interaction between *WRKYs* and *VQ* genes in grapevine compared to *Arabidopsis*. Nevertheless, this observation must be considered with due caution, considering the analysis was performed in one third of the gene predictions and in the promoter region longer than those ones considered in the present study.

### Expression analysis of *VvVQ* genes in various grapevine tissues at different developmental stages

**Figure 3:**
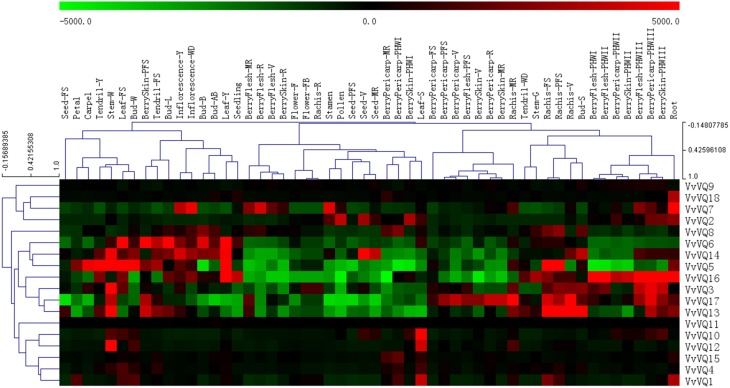
**Expression profile of 18 grapevine *VQ* genes in various tissues and at different developmental stages**. Expression data were normalized based on the mean expression value of each gene in all tissues/organs. Different organs/tissues are displayed vertically above each column. Gene names are displayed to the right of each row. Black boxes indicate a low variation with respect to the overall mean value of expression, green boxes indicate a fold decrease, and red boxes indicate a fold increase with respect to the mean value. Samples and genes were hierarchically clustered based on the average Pearson's distance. Abbreviations indicating developmental stages are as follows: FS, fruit set; PFS, post fruit set; V, véraison; MR, mid- ripening; R, ripening; Bud-L, latent bud; Bud-W, winter bud; Bud-S, bud swell; Bud-B, Bud burst; Bud-AB, bud after burst; Inflorescence-Y, young inflorescence with single flowers separated; Inflorescence-WD, well developed inflorescence; Flower-FB, flowering begins; Flower-F, flowering; Tendril-Y, young tendril; Tendril-WD, well developed tendril; Tendril-FS, mature tendril; Leaf-Y, young leaf; Leaf-FS, mature leaf; Leaf-S, senescing leaf; Stem-G, green stem; and Stem-W, woody stem.

Using an expression atlas of *V. vinifera* cv. Corvina (Fasoli et al., 2012), it was possible to investigate patterns of expression of all the predicted coding members of the *VvVQ* gene family in various tissues and at different developmental stages. All *VvVQ* genes had corresponding probes on the NimbleGen array. Figure 3 reports a graphical representation of the expression pattern of each *VvVQ* gene where both genes and samples were ordered based on a hierarchical clustering analysis. HCL tree based on gene leaves indicated a first clear difference between a group of *VvVQ* genes (*VvVQ8*, −*6*, −*14*, −*5*, −*16*, −*3*, −*17*, and −*13*) characterized by a marked modulation of expression in different tissues analyzed and the remaining genes, which showed much lower variability in terms of gene induction and/or repression. Among these were *VvVQ9* and *VvVQ11*, which did not show any variation in expression within all samples analyzed. This observation could be explained by the fact that both *VvVQ9* and *VvVQ11* have different roles in other developmental processes, tissues, or stress responses than those analyzed, or alternatively by a technical problem with the process of hybridizations in the array. *VvVQ18* is a particularly interesting gene because it is the only one whose expression appears to be limited to one specific organ: roots. To our knowledge, there is no information about a possible role of VQ proteins in root development. Nevertheless, it is well documented that there is a role for WRKY TFs in trichome development (Johnson et al., 2002). In a recent study aimed at characterizing the *WRKY* gene family in grapevine, Wang et al. (2014) identified several *VvWRKY* genes expressed in roots and, among them, *VvWRKY53* appeared exclusively expressed in this organ. *VvWRKY53* belongs to the group II-b and, based on previous Y2H assays performed by Cheng et al. (2012), it is not supposed to interact with VQ proteins. In any case, the possibility of an interaction between VQ proteins and WRKY TF belonging to groups other than I and II-c cannot be excluded *a priori* without any functional analysis. Other *WRKYs* highly expressed in roots are good candidates to interact with VvVQ18, such as *VvWRKY45* and *VvWRKY16*, both belonging to group I, and *VvWRKY43* which belongs to group II-c.

Together with *VvVQ18*, other *VQ* genes showed a quite specific expression in several tissues. Among these was *VvVQ1*, which similarly to *VvVQ18* was induced in roots, but also in senescing leaves (Leaf-S) and rachis prior to véraison (Rachis-FS and -PFS). *VvVQ10* and -*12* transcripts accumulated mainly in senescing leaves, even if limited to *VvVQ12*, a remarkable induction was also detected in winter stem (Stem -W). No other *VvVQs* (apart from *VvVQ2*, *−4*, and *−6* which showed a low induction) were strongly induced in senescing leaves. A role for VQ proteins in senescence has not been described before. Nevertheless, several WRKY proteins are known to regulate developmental processes such as leaf senescence (Robatzek and Somssich, 2002; Miao et al., 2004) and, considering the role of VQ proteins as WRKY co-factors, the hypothesis that some members of this gene family are involved also in these processes is far than unlikely. Leaf senescence is an active and highly regulated process that involves an integrated response of leaf cells to age information and other internal and environmental signals and is accompanied by a decreased expression of genes related to photosynthesis and protein synthesis and an increase in the expression of hundreds of senescence-associated genes (Espinoza et al., 2007). Among these are a number of pathogenesis-related (PR) genes and it was proposed that the senescence program might have incorporated features of the pathogen-defense response to protect the senescing leaf against opportunistic pathogens (Quirino et al., 2000). This would be in agreement with the well-documented role of several VQ proteins, in combination with WRKY TFs in the response to biotic stresses. Alternatively, the induction of *VvVQ* genes in senescing leaves may simply be a consequence of changes in the levels of various phytohormones, including SA and ETH, which are known to play an important role in regulating leaf senescence. This was also true in the present study and in a previous one (Cheng et al., 2012). SA- and ETH- responsive elements were found in the promoter regions of *VvVQ10* and *VvVQ12*, respectively.

Among those genes showing a higher level of variation within the Corvina expression atlas were *VvVQ2* and *VvVQ5*, which were highly expressed in pollen and carpel tissues, respectively. Some other *VvVQ* genes showed tissue specific expression pattern: 4 *VvVQs* (*VvVQ5*, −*6*, −*14*, and −*16*) had higher levels of expression in early leaf developmental stages (Y), even if a remarkable induction was also detectable in other tissues. Some *VvVQ* genes were more strictly associated with berry development. *VvVQ16*, for example, exhibited higher expression levels in berry flesh, berry pericarp, and berry skin during post-harvest withering (PHWII, PHWIII), while *VvVQ17* had higher expression in berry pericarp and berry skin at the earliest stages of development before ripening commenced (PF, V, MR) and post-harvest withering (PHWIII). Again, as postulated about the senescence process, many WRKY TFs were described to have a role in berry developmental processes and it is not surprising to find several *VvVQ* genes thatare expressed in developing berries. Based on the analysis performed by Wang et al. (2014), a strong induction of three WRKY TFs, namely *VvWRKY14*, −*19*, and −*52* was detected in developing berries. Interestingly, these WRKY TFs all belong to the group II-c, which is one of the two WRKY subfamilies able to interact with VQ proteins (Cheng et al., 2012).

To better understand *VvVQ* genes expression patterns, genes that showed differential expression during various tissues development were analyzed. The expression levels of each tissue at the first development stage were normalized (Figure S6). Genes listed above or below the specified tissue or stage of development indicated that they were up-regulated or down-regulated, respectively. The data indicated that *VvVQ* genes were expressed in a wide array of tissues and/or at specific stages of development. Up regulation of *VvVQ* genes mainly occurred in maturity and post-harvest stages of the berry pericarp, bud dormancy, and during stem and leaf development. Down regulation of many *VvVQ* genes was also evident in post-harvest berry pericarp tissues, berry skin (maturity), and during rachis development.

### Network co-expression analysis between *VvVQs* and group I and II-c *WRKY* TFs

**Figure 4:**
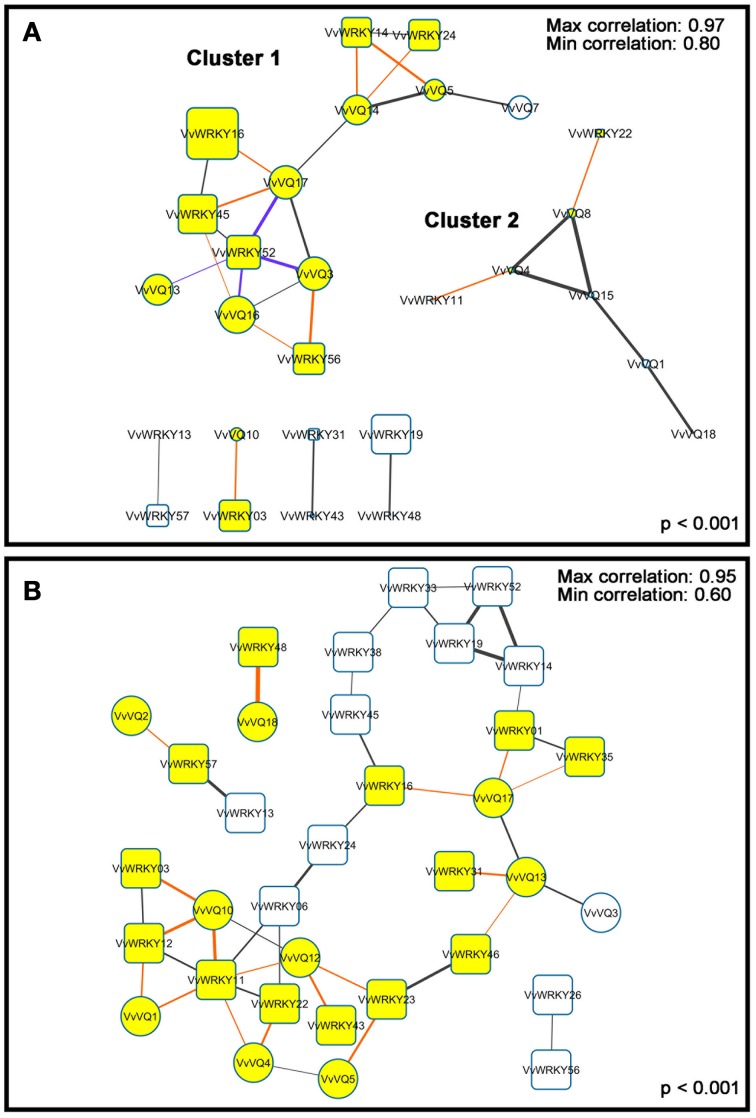
**Co-expression network of grapevine VQ motif-containing genes and *WRKY* genes according to a *p* < 0.001**. Thicker lines indicate more correlated genes. Blue and red lines, indicate negative and positive co-expression between a VQ motif-containing gene and a *WRKY* gene. *VQ-WRKY* correlated genes are showed in yellow. **(A)** Correlation network of PHW berries (Fasoli et al., 2012). The correlation coefficients of co-expression is between 0.8 and 0.97. Larger squares indicate more expressed genes. **(B)** Correlation network of all tissues described by Fasoli et al. (2012) except for PHW berries. The correlation coefficients of co-expression is between 0.6 and 0.95.

Previous studies have shown that 14 of the 34 *Arabidopsis* VQ motif-containing proteins can interact physiologically with WRKY proteins and that most of them have more than one WRKY partner (Andreasson et al., 2005; Wang et al., 2010a; Lai et al., 2011; Cheng et al., 2012; Hu et al., 2013). To gain information about hypothetical interactions between WRKY TFs and VQ proteins in grapevines, we performed a correlation analysis of expression of both *VvVQs* and *VvWRKYs* in the Corvina expression atlas. In order to simplify the network patterns, only the WRKY TFs belonging to group I and II-c, previously demonstrated to interact with the VQs in *Arabidopsis* (Cheng et al., 2012), were considered in the analysis. Two distinct datasets were used, one including the expression data in all tissues analyzed in the Corvina atlas (Fasoli et al., 2012) except the post-harvest withering, and the other one encompassing only berry pericarp, flesh and skin that were subjected to the PHW process. As reported by Vannozzi et al. (2012), the process of post-harvest berry drying (berry withering) involves harvesting of ripe grapevines, allowing them to dry over a period of 3 months in a naturally ventilated room. Its primary purpose is to alter berry quality characteristics and increase the concentration of simple sugars in the production of dessert and fortified wines typical of the Valpolicella region in Italy. However, the drying of harvested grapevines in this way results in a loss of over 30% of their weight through evaporation during this post-harvest treatment (Zamboni et al., 2008) and the speed of water loss induced by withering or other post-harvest techniques imposes a significant water stress on the berries (Corso et al., 2013). In the light of this information, the PHW process can be considered more as a “stress treatment” than a natural condition and the analysis restricted to these stages allowed us to compare the expression of both *WRKYs* and *VQs* under this “stress” condition and to assess putative transcriptional relationships. A first general observation was that no similar gene clusters (i.e., co-expressed *VQ* and *WRKY* genes) were found in the two considered correlation networks, highlighting strong specificity of *VQs* and *WRKYs* expression upon water stress in grapevine. These results were not unexpected, considering that several recent studies suggested a pivotal role of WRKYs in regulating osmotic stress responses in several species, such as *Arabidopsis*, rice (Rushton et al., 2012; Tripathi et al., 2014) and grapevine (Corso, personal communication). PHW correlation networks (Figure 4) divided *VQ* and *WRKY* genes into two main clusters characterized by high co-expression between *WRKY* and *VQ* genes (*r* > 0.89). In Figure 4A, Cluster 1 was characterized by the presence of co-expressed transcripts that showed very high expression values, whereas cluster 2 was composed of genes showing a significantly lower expression. In addition, a third cluster formed only by *VvVQ10* and *VvWRKY03* was also found. It is noteworthy that in PHW conditions, negative correlations were observed only for *VvWRKY52* with *VvVQ3* (r = −0.96), *VvVQ13* (r = −0.90), *VvVQ16* (r = −0.93) and *VvVQ17* (r = −0.96). As reported by Chi et al. (2013) a single WRKY protein belonging to Group I or II-c can potentially interact with several VQ proteins differentially affecting DNA-binding specificity or other properties of the interacting WRKY. Moreover, this interaction can also be linked to a negative regulation of the TF activities (Qiu et al., 2008). The co-expression of VvWRKY52 and above mentioned *VvVQ* transcripts poses the basis for further examinations aimed at determining if a real physical interaction exists between candidate partners.

Among *VQs* in the PHW subset, the gene with the higher average expression was *VvVQ16* (intensity: 9998), which was anti-correlated with *VvWRKY52* as described above, and co-expressed with *VvWRKY56* and *VvWRKY45*, orthologues of *AtWKY20* and *AtWKY58*, respectively (Figure 4). Interestingly, two different studies demonstrated that the *Arabidopsis* orthologues of *VvWRKY52* (*AtWRKY75*) and of *VvWRKY45* (*AtWRKY58*) are positive and negative regulators of plant responses and defense mechanisms to biotic stress (Balbi and Devoto, 2008; Encinas-Villarejo et al., 2009). In withering, *VvWRKY52* was repressed and *VvWRKY45* was induced, supporting the hypothesis of their involvement in response to stress and indicating that in grapevine diverse mechanisms are related to the kind of stress imposed, with the induction of different WRKYs TFs upon biotic or abiotic stresses.

In recent years, one of the most studied WRKY TFs has been *AtWRKY33*, which was reported to be significantly induced by osmotic and oxidative stresses (Jiang and Deyholos, 2009). *VvWRKY24* (average expression: 4471) and *VvWRKY16* (average expression: 23064), both orthologues of *AtWRKY33* (Wang et al., 2014) were strongly correlated (*r* = 0.91) with *VvVQ14* and *VvVQ17*, respectively, showing an increased expression from PHWI to PHWIII (Figure 3). As observed for *VvWRKY52* and correlated VvVQ transcripts, a functional analysis is mandatory to validate a cooperation of VvVQ14/17 andVvWRKY25/16 inmediating the abiotic stress responses in *Vitis*. The other dataset (Figure 4B) contains all of the grapevine organs and tissues described by Fasoli et al. (2012) except PHW berries. Correlation network analysis conducted on these data showed significant correlations (FDR < 0.05 and *r* > 0.6) for 10 *VQ* and 24 *WRKY* transcripts. Among these, *VvVQ18* and *VvWRKY48* were the genes showing the highest correlation values (*r* = 0.95). These genes are both characterized by a high and specific expression in roots as previously described (Figure 3). Another interesting gene was *VvVQ10*, that is mainly expressed in senescing leaves and co-expressed with *VvWRKY03, −11*, and −*12* of group II-c (Wang et al., 2014). Phylogenetic analysis revealed that *VvWRKY03* is similar to *AtWRKY75*, while *VvWRKY11* and −*12* shows sequence similarity with *AtWRKY51*. Guo et al. (2004) suggested that *AtWRKY75* and −*51* are senescence-specific genes and, only for the first one, is leaf senescence significantly delayed in related *Arabidopsis* antisense lines.

Interestingly, some water stress-induced *WRKY* genes were co-expressed with one another (Figure 4). Despite accumulating data suggesting that some WRKY proteins belonging to groups II-a, -b, -d and III function in the same pathway in response to different biotic and abiotic stresses (Chi et al., 2013), it is possible that these interactions may occur also between group I and II-c WRKYs TFs. Thus, further studies could also focus on determining whether different combinations of WRKY proteins cooperate with VQ motif-containing proteins in grapevine berries upon abiotic stresses.

### Expression profiles of grapevine *VQ* genes in response to different stress and exogenous hormone treatments

#### Stress treatments

**Figure 5:**
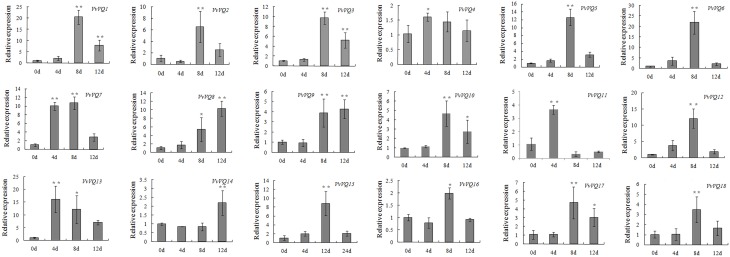
**Expression patterns of grapevine *VQ* genes in response to a drought treatment**. Quantitative RT-PCR was used to determine the level of expression of *VQ* genes in response to a drought treatment. The Y axis represents the level of relative gene expression while the X axis represents the time course over which samples were collected after water was withheld. The name of each *VvVQ* gene is provided in each graph. The values of *VvVQ* gene expression were normalized using the expression of a grapevine actin gene. Data points represent the mean of three independent biological replicates. The error bars indicate standard deviation. ^*^ indicates that the level of expression is significantly different from the value of the control (^*^P < 0.05, ^**^P < 0.01).

Increasing evidence indicates that *VQ* genes are associated with plant response to environmental stimuli and that the expression of *VQ* genes is enhanced by drought, pathogen inoculation, and treatment with hormones (Cheng et al., 2012; Kim et al., 2013). It has been reported that 22 rice *VQ* genes are up-regulated in response to drought treatment (Kim et al., 2013). The *Arabidopsis* VQ9 protein has been shown to act as a repressor of WRKY8 to maintain a balance in the activation of WRKY8-mediated signaling pathways that are involved in salinity stress tolerance (Hu et al., 2013). In our expression analysis, we identified few *VvVQ* genes, *VvVQ3*, −*16*, and −*17*, strictly related to dehydration and linked to the withering process (Figure 3). Anyway, it's worthwhile to note that, although involving a dehydration process, post-withering represents a very particular condition and is limited to berry tissues. In order to analyse more in detail the response of *VvVQ* genes to drought stress, the expression levels of all the 18 *VQ* genes were analyzed in leaves of Pinot Noir 40024 plants subjected to water deprivation. Transcript accumulation was measured by quantitative RT-PCR and is expressed as fold-change (FC) in Figure 5. The majority of *VvVQ* genes were found to be responsive to water stress. For four *VQs* (*VvVQ4, −7*, −*11*, and −*13*), a significant induction was detected 4 days after the imposition of the stress, 13 *VvVQs* were highly expressed from the 8th day and only *VvVQ14* showed an induction after the 12th day. *VvVQ1* and −*6* were the most responsive genes, with FC values higher than 20, while *VvVQ4* was the only gene very weakly modulated by the stress and with a FC < 2 and thus it can be considered not responsive to the water stress. *VvVQ8*, −*9*, and −*14* were the only genes shown to gradually increase their expression and showed a peak at 12 day with *VvVQ8* being the only one reaching FC values higher than 10. The up-regulation of the *VvVQ3*, −*16*, and −*17* under water stress conditions, their high expression levels in PHW berry tissues observed in the Corvina atlas (Figure 3) and the presence of MBS *cis*-elements involved in drought response in the promoter regions strongly suggest a role of these VQs in the response to water stress. As in Wang et al. (2014), the expression of some *VvWRKY* genes is induced by water deficit stress, such as *VvWRKY03*, *VvWRKY16*, and *VvWRKY52*. In the correlation network analysis on berry tissues undergoing the withering process, (Figure 4A) these genes were linked to *VvVQ10*, −*13*, −*16*, −*3*, and −*17*. However, the induction of both *VvWRKY52* and *VvVQ13*, −*16*, −*3*, and −*17* detected by qRT-PCR analysis is in contrast with the negative correlation observed in the network analysis. As reported in Table S3, the majority of *VvVQ* genes showing induction upon water stress presents at least one drought responsive MBS element within the first 2 Kb upstream the start codon. These data are in agreement with previous expression analyses performed in rice (Kim et al., 2013), where most *VQ* genes were demonstrated to be involved in the response to water stress.

**Figure 6:**
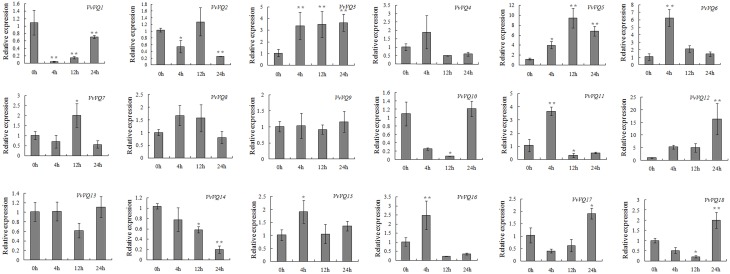
**Expression patterns of grapevine *VQ* genes in response to inoculation with *Erysiphe necator* (powdery mildew)**. Quantitative RT-PCR was used to determine the level of expression of *VQ* genes in response to inoculation with powdery mildew. Gene names are indicated in each graph. The values of *VvVQ* gene expression were normalized using the expression of a grapevine actin gene. Data points represent the mean of three independent biological replicates. The error bars indicate standard deviation. ^*^ indicates that the level of expression is significantly different from the value of the control (^*^P < 0.05, ^**^P < 0.01).

As demonstrated for the WRKY TFs, many VQ proteins of *Arabidopsis* and rice are involved in the plant response to biotic stresses (Cheng et al., 2012; Kim et al., 2013). In order to investigate the response of the whole *VvVQ* family in grapevine upon biotic stress, the expression patterns of all *VvVQ* transcripts was monitored by qRT-PCR also upon powdery mildew infection. As shown in Figure 6, 5 out of 18 genes (*VvVQ3*, −*5*, −*6*, −*11*, and −*12*) were significantly up-regulated upon powdery mildew infection. *VvVQ12* is the most responsive gene, increasing its expression across all profiles and reaching a peak at 24 h after inoculation with the FC value of approximately 15 in respect to the mock-inoculated samples. *VvVQ3* showed an up regulation at 4 h after inoculation and maintained the same level through the whole profile. The transcript abundance of *VvVQ5* in inoculated leaves peaked at 12 h after infection, whereas *VvVQ6* and *VvVQ11* were induced only at 4 h. The inoculation treatment with powdery mildew also caused an increase in the transcription levels of *VvVQ16* at 4 h, however, the expression level then dropped below the level of expression observed prior to inoculation. In contrast, the transcript abundance of *VvVQ17* and *VvVQ18* was initially decreased up to 12 h after inoculation, but then increased substantially at 24 h (Figure 6). The expression of *VvVQ1*, −*10*, and −*14*, despite having different patterns, showed a clear down-regulation upon infection. These analyses indicated that VvVQ TFs were implicated in the powdery mildew response, possibly through interaction with WRKY proteins. Involvement of both VQ and WRKY proteins in plant defense was already reported, showing that the *Arabidopsis* VQ proteins SIB1 and SIB2 act as activators of WRKY33, which in turn is involved in the plant defense against necrotrophic pathogens (Lai et al., 2011). Moreover, MKS1, a VQ protein that acts as a substrate for MAPK4 (MPK4) in *Arabidopsis*, interacts with WRKY25 and WRKY33 and thus may contribute to MAPK-regulated defense activation by coupling the kinase to specific WRKY TFs (Andreasson et al., 2005).

#### Hormone treatments

**Figure 7:**
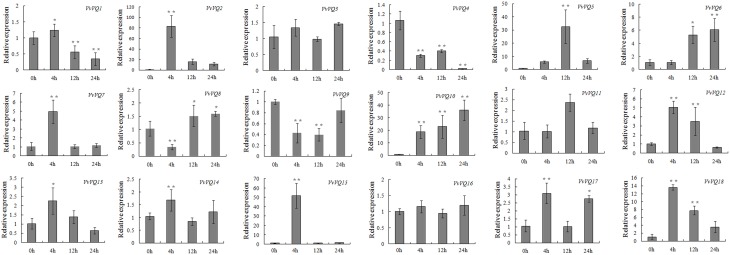
**Expression patterns of grapevine *VQ* genes in response to salicylic acid (SA) treatment**. Quantitative RT-PCR was used to determine the level of expression of *VQ* genes in response to the application of salicylic acid (SA). Gene names are indicated in each graph. The values of *VvVQ* gene expression were normalized using the expression of a grapevine actin gene. Data points represent the mean of three independent biological replicates. The error bars indicate standard deviation. ^*^ indicates that the level of expression is significantly different from the value of the control (^*^P < 0.05, ^**^P < 0.01).

SA and ETH mediate plant responses to biotic stress (Broekaert et al., 2006; Loake and Grant, 2007). To investigate their effect on the expression of the *VvVQ* genes, in the current study, quantitative RT-PCR was conducted on the 18 *VvVQ* genes in plants treated with these two hormones. As shown in Figure 7, the expression profiles after SA treatment appeared modulated for most of the *VvVQ* genes with the exception of *VvVQ3* and *VvVQ16*, which showed unaltered transcript levels. In particular, *VvVQ2*, −*5*, −*6*, −*10*, and −*18* were strongly up-regulated even if by varying degrees throughout the treatment. Whereas, *VvVQ4* and *VvVQ9*, were significantly down-regulated. *VvVQ7* and *VvVQ15* were significantly induced at 4 h after SA treatment and then declined to the normal level.

**Figure 8:**
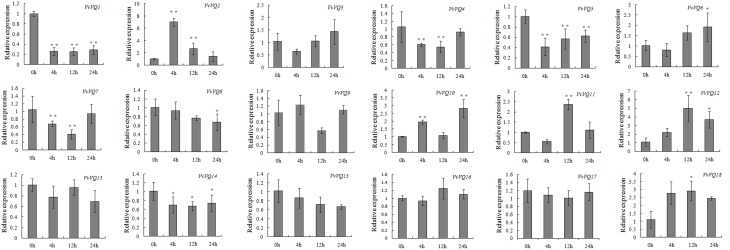
**Expression patterns of grapevine *VQ* genes in response to ethylene (ETH) treatment**. Quantitative RT-PCR was used to determine the level of expression of *VQ* genes in response to an application of ETH. Gene names are indicated in each graph. The values of *VvVQ* gene expression were normalized using the expression of a grapevine actin gene. Data points represent the mean of three independent biological replicates. The error bars indicate standard deviation. ^*^ indicates that the level of expression is significantly different from the value of the control (^*^P < 0.05, ^**^P < 0.01).

Analysis of transcript abundance in grapevine leaves treated exogenously with ETH, reported in Figure 8, indicated that *VvVQ2*, −*12*, and −*18* were the only *VvVQ* genes exhibiting higher levels of expression than that of the control, suggesting that in general the *VvVQs* were induced more by SA than ETH. For *VvVQ4* and *VvVQ7*, the expression patterns showed a very slight drop only at 4, −12 h after treatment, whereas the levels of *VvVQ13*, −*16*, −*15*, and -*17* remained unchanged. The expression of *VvVQ1*, −*5*, −*8*, and−*14* were significantly repressed throughout the experiment.

In general, 11 *VvVQ* genes presented similar expression patterns in both SA and ETH experiments, even if with different FCs. Among these, *VvVQ3*, −*9*, and −*16* were not modulated, indicating that they are probably not responsive to these hormones. Interestingly*, VvVQ5*, −*7*, −*8*, −*13*, −*15*, −*17*, and −*18* showed different responses to the hormones, being down-regulated or not modulated by the ETH treatment, but strongly induced by SA. Similar responses to SA and ETH hormones were also observed for many *VvWRKY* genes by Guo et al. (2014). Interestingly, among them was *VvWRKY44* of subgroup II-c (corresponding to *VvWRKY43*) (Wang et al., 2014), whose expression was induced by both SA and ETH (Guo et al., 2014), and highly correlated with *VvVQ12* in our analysis. *VvVQ12* was also up-regulated by both treatments (Figures 7, 8); it is conceivable that *VvWRKY43* and *VvVQ12* proteins cooperate in the response to SA and ETH. Our expression analysis of the *VvVQ* gene family after abiotic, biotic stresses and treatments by hormones together with the correlation analysis of gene expression provide useful information about the hypothetical roles of *VvVQ* genes in the regulation of developmental and defense mechanisms and about possible interactions with the WRKY proteins.

## Conclusions

Based on studies with *Arabidopsis*, it appears that plant VQ proteins primarily function as cofactors of WRKY proteins and play critical roles in regulating WRKY-mediated gene expression (Cheng et al., 2012). In the present study, the grapevine VQ gene family was examined and 18 *VvVQ* genes were identified. The phylogeny, compositional structure, and cellular location (based on homology with *VQ* genes in other species) of *VvVQ* genes were determined, providing a general overview of this multigenic family in grapevine. The expression of *VvVQ* genes in different tissues and different developmental stages was examined by using publicly available microarray data. Expression data, together with the correlation network analysis carried out with highly correlated *VQ* and *WRKY* genes, highlighted that *VvWRKY52* and *VQ3*, −*13*, −*16*, and −*17* were strongly anti-correlated in PHW conditions, suggesting that further analyses need to be conducted to determine whether the proteins encoded by these genes interact one another. Moreover, these data led to the hypotesis that *VvVQ14* and *VvVQ17* may cooperate with *VvWRKY25* and *VvWRKY16* to mediate abiotic stress responses in grapevines. In this study, qRT-PCR was used to analyse the expression of *VvVQ* transcripts in response to drought, powdery mildew inoculation, and treatment with SA and ETH. Tissue-specific and developmental-specific patterns of *VQ* gene expressions were observed in grapevine, as was the up- and down-regulation of specific *VvVQ* genes in response to biotic and abiotic stress treatments, and hormone treatments. Collectively, the data generated in this study of *VQ* genes in grapevine provided a useful resource for future functional studies.

### Conflict of interest statement

The authors declare that the research was conducted in the absence of any commercial or financial relationships that could be construed as a potential conflict of interest.

## Supplementary material

Table S1 **List of primers used to quantify *VvVQ* genes expression by qRT-PCR**.

Table S2 **Syntenic blocks of *VQ* genes between grapevine and *Arabidopsis* genomes**.

Table S3 **Promoter analysis of the grapevine *VQ* gene family**.

Figure S1 **Chromosomal location of *VQ* genes in grapevine**. The chromosomal location of grapevine *VQ* genes was determined based on the 12X V1 release of the PN40024 grapevine genome (http://genomes.cribi.unipd.it/). The distribution of *VvVQ* genes was drawn using MapInspect software.

Figure S2 **Alignment of VQ domain amino acid sequences of grapevine VQ proteins**. Multiple alignment of grapevine VQ domain amino acid sequences was performed using Muscle with default options.

Figure S3 **Phylogenetic tree of grapevine, *Arabidopsis* and rice VQ proteins obtained using a Bayesian-base method**.

Figure S4 **Chromosomal location of *VQ* genes in *Arabidopsis* indicating tandem and segmental duplications**. Diagram of the five chromosomes of Arabidopsis depicting the location and distribution of 34*VQ* genes. The segmental duplication genes were connected with a blue line. Tandem duplication genes were indicated with a pink vertical line.

Figure S5 **Syntenic blocks of *VQ* genes (indicated in red) between the grapevine and *Arabidopsis* genomes**. Arrows represent gene loci in the grapevine and *Arabidopsis* chromosomes. Blue arrows represent duplicated loci in a syntenic block. The data were obtained from the Plant Genome Duplication Database, and syntenic blocks containing grapevine *VQ* genes were illustrated.

Figure S6 **Differential expression of *VvVQ* genes in various tissues and at different developmental stages based on microarray analysis**. The expression levels of the first development stage of each tissue were set at a value of “1” to determine up and down regulation at subsequent stages of development. Tissues containing only one phase were not considered. The genes above and below the colored tissue bar indicated the up-regulated and down-regulated genes, respectively, at this developmental stage. Genes were considered to be differentially expressed if the fold change (up- or down- regulated) was greater than two.

## Abbreviations

*VvVQ*: Grapevine *VQ*
SA: Salicylic acid
ETH: Ethylene
TF: Transcription factor
qRT-PCR: Quantitative reverse transcription polymerase chain reaction
MEME: Multiple Expectation Maximization for Motif Elicitation
aa: amino acid/s.

## References

[B1] Andreasson E. Jenkins T. Brodersen P. Thorgrimsen S. Petersen N. H. Zhu S.. (2005). The MAP kinase substrate MKS1 is a regulator of plant defense responses. EMBO J. 24, 2579–2589. 10.1038/sj.emboj.7600737 15990873 PMC1176463

[B2] Bailey T. L. Boden M. Buske F. A. Frith M. Grant C. E. Clementi L.. (2009). MEME SUITE: tools for motif discovery and searching. Nucleic Acids Res. 37, W202–W208. 10.1093/nar/gkp335 19458158 PMC2703892

[B3] Balbi V. Devoto A. (2008). Jasmonate signalling network in *Arabidopsis thaliana*: crucial regulatory nodes and new physiological scenarios. New Phytol. 177, 301–318. 10.1111/j.1469-8137.2007.02292.x 18042205

[B4] Bowers J. E. Chapman B. A. Rong J. K. Paterson A. H. (2003). Unravelling angiosperm genome evolution by phylogenetic analysis of chromosomal duplication events. Nature 422, 433–438. 10.1038/nature01521 12660784

[B5] Broekaert W. F. Delaure S. L. De Bolle M. F. Cammue B. P. (2006). The role of ethylene in host-pathogen interactions. Annu. Rev. Phytopathol. 44, 393–416. 10.1146/annurev.phyto.44.070505.143440 16602950

[B6] Chen H. Lai Z. Shi J. Xiao Y. Chen Z. Xu X. (2010). Roles of arabidopsis WRKY18, WRKY40 and WRKY60 transcription factors in plant responses to abscisic acid and abiotic stress. BMC Plant Biol. 10:281. 10.1186/1471-2229-10-281 21167067 PMC3023790

[B7] Cheng Y. Zhou Y. Yang Y. Chi Y. J. Zhou J. Chen J. Y.. (2012). Structural and functional analysis of VQ motif-containing proteins in Arabidopsis as interacting proteins of WRKY transcription factors. Plant Physiol. 159, 810–825. 10.1104/pp.112.196816 22535423 PMC3375943

[B8] Chi Y. Yang Y. Zhou Y. Zhou J. Fan B. Yu J.-Q.. (2013). Protein–protein interactions in the regulation of WRKY transcription factors. Mol. Plant. 6, 287–300. 10.1093/mp/sst026 23455420

[B9] Corso M. Ziliotto F. Rizzini F. M. Teo G. Cargnello G. Bonghi C. (2013). Sensorial, biochemical and molecular changes in Raboso Piave grape berries applying “Double Maturation Raisonnée” and late harvest techniques. Plant Sci. 208, 50–57. 10.1016/j.plantsci.2013.03.010 23683929

[B10] Daraselia N. D. Tarchevskaya S. Narita J. O. (1996). The promoter for tomato 3-hydroxy-3-methylglutaryl coenzyme A reductase gene 2 has unusual regulatory elements that direct high-level expression. Plant Physiol. 112, 727–733. 10.1104/pp.112.2.727 8883384 PMC157997

[B11] Dong J. Chen C. Chen Z. (2003). Expression profiles of the Arabidopsis WRKY gene superfamily during plant defense response. Plant Mol. Biol. 51, 21–37. 10.1023/A:1020780022549 12602888

[B12] Edgar R. C. (2004). MUSCLE: multiple sequence alignment with high accuracy and high throughput. Nucleic Acids Res. 32, 1792–1797. 10.1093/nar/gkh340 15034147 PMC390337

[B13] Encinas-Villarejo S. Maldonado A. M. Amil-Ruiz F. De Los Santos B. Romero F. Pliego-Alfaro F.. (2009). Evidence for a positive regulatory role of strawberry (Fragaria×ananassa) Fa WRKY1 and Arabidopsis At WRKY75 proteins in resistance. J. Exp. Bot. 60, 3043–3065. 10.1093/jxb/erp152 19470657

[B14] Espinoza C. Medina C. Somerville S. Arce-Johnson P. (2007). Senescence-associated genes induced during compatible viral interactions with grapevine and Arabidopsis. J. Exp. Bot. 58, 3197–3212. 10.1093/jxb/erm165 17761729

[B15] Eulgem T. Rushton P. J. Robatzek S. Somssich I. E. (2000). The WRKY superfamily of plant transcription factors. Trends Plant Sci. 5, 199–206. 10.1016/S1360-1385(00)01600-9 10785665

[B16] Fasoli M. Dal Santo S. Zenoni S. Tornielli G. B. Farina L. Zamboni A. (2012). The grapevine expression atlas reveals a deep transcriptome shift driving the entire plant into a maturation program. Plant Cell 24, 3489–3505. 10.1105/tpc.112.100230 22948079 PMC3480284

[B17] Freitas F. Z. Bertolini M. C. (2004). Genomic organization of the Neurospora crassa gsn gene: possible involvement of the STRE and HSE elements in the modulation of transcription during heat shock. Mol. Genet. Genomics. 272, 550–561. 10.1007/s00438-004-1086-5 15558319

[B18] Gaffney T. Friedrich L. Vernooij B. Negrotto D. Nye G. Uknes S.. (1993). Requirement of salicylic Acid for the induction of systemic acquired resistance. Science 261, 754–756. 10.1126/science.261.5122.754 17757215

[B19] Guo C. Guo R. Xu X. Gao M. Li X. Song J.. (2014). Evolution and expression analysis of the grape (*Vitis vinifera* L.) WRKY gene family. J. Exp. Bot. 65, 1513–1528. 10.1093/jxb/eru007 24510937 PMC3967086

[B20] Guo Y. Cai Z. Gan S. (2004). Transcriptome of Arabidopsis leaf senescence. Plant Cell Environ. 27, 521–549. 10.1111/j.1365-3040.2003.01158.x

[B21] Hu Y. Chen L. Wang H. Zhang L. Wang F. Yu D. (2013). Arabidopsis transcription factor WRKY8 functions antagonistically with its interacting partner VQ9 to modulate salinity stress tolerance. Plant J. 74, 730–745. 10.1111/tpj.12159 23451802

[B22] Huelsenbeck J. P. Ronquist F. (2001). MRBAYES: bayesian inference of phylogeny. Bioinformatics 17, 754–755. 10.1093/bioinformatics/17.8.754 11524383

[B23] Jaillon O. Aury J. M. Noel B. Policriti A. Clepet C. Casagrande A.. (2007). The grapevine genome sequence suggests ancestral hexaploidization in major angiosperm phyla. Nature 449, 463–467. 10.1038/nature06148 17721507

[B24] Jiang W. Yu D. (2009). Arabidopsis WRKY2 transcription factor mediates seed germination and postgermination arrest of development by abscisic acid. BMC Plant Biol. 9:96. 10.1186/1471-2229-9-96 19622176 PMC2719644

[B25] Jiang Y. Deyholos M. (2009). Functional characterization of Arabidopsis NaCl-inducible WRKY25 and WRKY33 transcription factors in abiotic stresses. Plant Mol. Biol. 69, 91–105. 10.1007/s11103-008-9408-3 18839316

[B26] Johnson C. S. Kolevski B. Smyth D. R. (2002). TRANSPARENT TESTA GLABRA2, a trichome and seed coat development gene of Arabidopsis, encodes a WRKY transcription factor. Plant Cell 14, 1359–1375. 10.1105/tpc.001404 12084832 PMC150785

[B27] Journot-Catalino N. Somssich I. E. Roby D. Kroj T. (2006). The transcription factors WRKY11 and WRKY17 act as negative regulators of basal resistance in *Arabidopsis thaliana*. Plant Cell 18, 3289–3302. 10.1105/tpc.106.044149 17114354 PMC1693958

[B28] Kim D. Y. Kwon S. I. Choi C. Lee H. Ahn I. Park S. R.. (2013). Expression analysis of rice VQ genes in response to biotic and abiotic stresses. Gene 529, 208–214. 10.1016/j.gene.2013.08.023 23958655

[B29] Kim J. K. Cao J. Wu R. (1992). Regulation and interaction of multiple protein factors with the proximal promoter regions of a rice high pI alpha-amylase gene. Mol. Gen. Genet. 232, 383–393. 10.1007/BF00266241 1375314

[B30] Kim K. C. Lai Z. Fan B. Chen Z. (2008). Arabidopsis WRKY38 and WRKY62 transcription factors interact with histone deacetylase 19 in basal defense. Plant Cell 20, 2357–2371. 10.1105/tpc.107.055566 18776063 PMC2570728

[B31] Lai Z. Li Y. Wang F. Cheng Y. Fan B. Yu J. Q.. (2011). Arabidopsis sigma factor binding proteins are activators of the WRKY33 transcription factor in plant defense. Plant Cell 23, 3824–3841. 10.1105/tpc.111.090571 21990940 PMC3229152

[B32] Lai Z. Vinod K. Zheng Z. Fan B. Chen Z. (2008). Roles of Arabidopsis WRKY3 and WRKY4 transcription factors in plant responses to pathogens. BMC Plant Biol. 8:68. 10.1186/1471-2229-8-68 18570649 PMC2464603

[B33] Li S. Fu Q. Huang W. Yu D. (2009). Functional analysis of an Arabidopsis transcription factor WRKY25 in heat stress. Plant Cell Rep. 28, 683–693. 10.1007/s00299-008-0666-y 19125253

[B34] Liu Q. Wang H. Zhang Z. Wu J. Feng Y. Zhu Z. (2009). Divergence in function and expression of the NOD26-like intrinsic proteins in plants. BMC Genomics 10:313. 10.1186/1471-2164-10-313 19604350 PMC2726226

[B35] Liu X. Wang X. Pang Y. Liang J. Liu S. Sun X.. (2006). [Molecular cloning and characterization of a novel WRKY gene from Brassica chinensis]. Mol. Biol. (Mosk) 40, 816–824. 10.1134/s0026893306050074 17086982

[B36] Loake G. Grant M. (2007). Salicylic acid in plant defence–the players and protagonists. Curr. Opin. Plant Biol. 10, 466–472. 10.1016/j.pbi.2007.08.008 17904410

[B37] Lois R. Dietrich A. Hahlbrock K. Schulz W. (1989). A phenylalanine ammonia-lyase gene from parsley: structure, regulation and identification of elicitor and light responsive cis-acting elements. EMBO J. 8, 1641–1648. 2767049 10.1002/j.1460-2075.1989.tb03554.x PMC401003

[B38] Luo M. Dennis E. S. Berger F. Peacock W. J. Chaudhury A. (2005). MINISEED3 (MINI3), a WRKY family gene, and HAIKU2 (IKU2), a leucine-rich repeat (LRR) KINASE gene, are regulators of seed size in Arabidopsis. Proc. Natl. Acad. Sci. U.S.A. 102, 17531–17536. 10.1073/pnas.0508418102 16293693 PMC1297679

[B39] Lyons E. Pedersen B. Kane J. Alam M. Ming R. Tang H.. (2008). Finding and comparing syntenic regions among Arabidopsis and the outgroups papaya, poplar, and grape: CoGe with rosids. Plant Physiol. 148, 1772–1781. 10.1104/pp.108.124867 18952863 PMC2593677

[B40] Manzara T. Carrasco P. Gruissem W. (1991). Developmental and organ-specific changes in promoter DNA-protein interactions in the tomato rbcS gene family. Plant Cell 3, 1305–1316. 10.1105/tpc.3.12.1305 1840899 PMC160093

[B41] Menkens A. E. Schindler U. Cashmore A. R. (1995). The G-box: a ubiquitous regulatory DNA element in plants bound by the GBF family of bZIP proteins. Trends Biochem. Sci. 20, 506–510. 10.1016/S0968-0004(00)89118-5 8571452

[B42] Miao Y. Laun T. Zimmermann P. Zentgraf U. (2004). Targets of the WRKY53 transcription factor and its role during leaf senescence in Arabidopsis. Plant Mol. Biol. 55, 853–867. 10.1007/s11103-005-2142-1 15604721

[B43] Pastuglia M. Roby D. Dumas C. Cock J. M. (1997). Rapid induction by wounding and bacterial infection of an S gene family receptor-like kinase gene in Brassica oleracea. Plant Cell 9, 49–60. 10.1105/tpc.9.1.49 9014364 PMC156900

[B44] Qiu J. L. Fiil B. K. Petersen K. Nielsen H. B. Botanga C. J. Thorgrimsen S.. (2008). Arabidopsis MAP kinase 4 regulates gene expression through transcription factor release in the nucleus. EMBO J. 27, 2214–2221. 10.1038/emboj.2008.147 18650934 PMC2519101

[B45] Quirino B. F. Noh Y.-S. Himelblau E. Amasino R. M. (2000). Molecular aspects of leaf senescence. Trends Plant Sci. 5, 278–282. 10.1016/S1360-1385(00)01655-1 10871899

[B46] Ramamoorthy R. Jiang S. Y. Kumar N. Venkatesh P. N. Ramachandran S. (2008). A comprehensive transcriptional profiling of the WRKY gene family in rice under various abiotic and phytohormone treatments. Plant Cell Physiol. 49, 865–879. 10.1093/pcp/pcn061 18413358

[B47] Robatzek S. Somssich I. E. (2002). Targets of AtWRKY6 regulation during plant senescence and pathogen defense. Genes Dev. 16, 1139–1149. 10.1101/gad.222702 12000796 PMC186251

[B48] Rouster J. Leah R. Mundy J. Cameron-Mills V. (1997). Identification of a methyl jasmonate-responsive region in the promoter of a lipoxygenase 1 gene expressed in barley grain. Plant J. 11, 513–523. 10.1046/j.1365-313X.1997.11030513.x 9107039

[B49] Rushton D. L. Tripathi P. Rabara R. C. Lin J. Ringler P. Boken A. K.. (2012). WRKY transcription factors: key components in abscisic acid signalling. Plant Biotechnol. J. 10, 2–11. 10.1111/j.1467-7652.2011.00634.x 21696534

[B50] Rushton P. J. Torres J. T. Parniske M. Wernert P. Hahlbrock K. Somssich I. E. (1996). Interaction of elicitor-induced DNA-binding proteins with elicitor response elements in the promoters of parsley PR1 genes. EMBO J. 15, 5690–5700. 8896462 PMC452313

[B51] Saeed A. I. Bhagabati N. K. Braisted J. C. Liang W. Sharov V. Howe E. A.. (2006). TM4 microarray software suite. Methods Enzymol. 411, 134–193. 10.1016/S0076-6879(06)11009-5 16939790

[B52] Shang Y. Yan L. Liu Z. Q. Cao Z. Mei C. Xin Q.. (2010). The Mg-chelatase H subunit of Arabidopsis antagonizes a group of WRKY transcription repressors to relieve ABA-responsive genes of inhibition. Plant Cell 22, 1909–1935. 10.1105/tpc.110.073874 20543028 PMC2910980

[B53] Shannon P. Markiel A. Ozier O. Baliga N. S. Wang J. T. Ramage D.. (2003). Cytoscape: a software environment for integrated models of biomolecular interaction networks. Genome Res. 13, 2498–2504. 10.1101/gr.1239303 14597658 PMC403769

[B54] Simpson S. D. Nakashima K. Narusaka Y. Seki M. Shinozaki K. Yamaguchi-Shinozaki K. (2003). Two different novel cis-acting elements of erd1, a clpA homologous Arabidopsis gene function in induction by dehydration stress and dark-induced senescence. Plant J. 33, 259–270. 10.1046/j.1365-313X.2003.01624.x 12535340

[B55] Suttipanta N. Pattanaik S. Kulshrestha M. Patra B. Singh S. K. Yuan L. (2011). The transcription factor CrWRKY1 positively regulates the terpenoid indole alkaloid biosynthesis in Catharanthus roseus. Plant Physiol. 157, 2081–2093. 10.1104/pp.111.181834 21988879 PMC3327198

[B56] Tamura K. Peterson D. Peterson N. Stecher G. Nei M. Kumar S. (2011). MEGA5: molecular evolutionary genetics analysis using maximum likelihood, evolutionary distance, and maximum parsimony methods. Mol. Biol. Evol. 28, 2731–2739. 10.1093/molbev/msr121 21546353 PMC3203626

[B57] Tang H. Wang X. Bowers J. E. Ming R. Alam M. Paterson A. H. (2008a). Unraveling ancient hexaploidy through multiply-aligned angiosperm gene maps. Genome Res. 18, 1944–1954. 10.1101/gr.080978.108 18832442 PMC2593578

[B58] Tang H. B. Bowers J. E. Wang X. Y. Ming R. Alam M. Paterson A. H. (2008b). Perspective - Synteny and collinearity in plant genomes. Science 320, 486–488. 10.1126/science.1153917 18436778

[B60] Tripathi P. Rabara R. Rushton P. (2014). A systems biology perspective on the role of WRKY transcription factors in drought responses in plants. Planta 239, 255–266. 10.1007/s00425-013-1985-y 24146023

[B61] Vannozzi A. Dry I. B. Fasoli M. Zenoni S. Lucchin M. (2012). Genome-wide analysis of the grapevine stilbene synthase multigenic family: genomic organization and expression profiles upon biotic and abiotic stresses. BMC Plant Biol. 12:130 10.1186/1471-2229-12-130 22863370 PMC3433347

[B62] Velasco R. Zharkikh A. Troggio M. Cartwright D. A. Cestaro A. Pruss D.. (2007). A high quality draft consensus sequence of the genome of a heterozygous grapevine variety. PLoS ONE 2:e1326. 10.1371/journal.pone.0001326 18094749 PMC2147077

[B63] Vitulo N. Forcato C. Carpinelli E. C. Telatin A. Campagna D. D'Angelo M.. (2014). A deep survey of alternative splicing in grape reveals changes in the splicing machinery related to tissue, stress condition and genotype. BMC Plant Biol. 14:99. 10.1186/1471-2229-14-99 24739459 PMC4108029

[B64] Wang A. Garcia D. Zhang H. Feng K. Chaudhury A. Berger F.. (2010a). The VQ motif protein IKU1 regulates endosperm growth and seed size in Arabidopsis. Plant J. 63, 670–679. 10.1111/j.1365-313X.2010.04271 20545893

[B65] Wang H. Avci U. Nakashima J. Hahn M. G. Chen F. Dixon R. A. (2010b). Mutation of WRKY transcription factors initiates pith secondary wall formation and increases stem biomass in dicotyledonous plants. Proc. Natl. Acad. Sci. U.S.A. 107, 22338–22343. 10.1073/pnas.1016436107 21135241 PMC3009815

[B66] Wang M. Vannozzi A. Wang G. Liang Y.-H. Tornielli G. B. Zenoni S. (2014). Genome and transcriptome analysis of the grapevine (*Vitis vinifera* L.) WRKY gene family. Horticult. Res. 1:16 10.1038/hortres.2014.16 PMC4596322 26504535

[B67] Washida H. Wu C. Y. Suzuki A. Yamanouchi U. Akihama T. Harada K.. (1999). Identification of cis-regulatory elements required for endosperm expression of the rice storage protein glutelin gene GluB-1. Plant Mol. Biol. 40, 1–12. 10.1023/A:1026459229671 10394940

[B68] Xu X. Chen C. Fan B. Chen Z. (2006). Physical and functional interactions between pathogen-induced Arabidopsis WRKY18, WRKY40, and WRKY60 transcription factors. Plant Cell 18, 1310–1326. 10.1105/tpc.105.037523 16603654 PMC1456877

[B69] Xu Y. Chang P. Liu D. Narasimhan M. L. Raghothama K. G. Hasegawa P. M.. (1994). Plant defense genes are synergistically induced by ethylene and methyl jasmonate. Plant Cell 6, 1077–1085. 10.1105/tpc.6.8.1077 12244267 PMC160502

[B70] Yamaguchi-Shinozaki K. Shinozaki K. (1993). Arabidopsis DNA encoding two desiccation-responsive rd29 genes. Plant Physiol. 101, 1119–1120. 10.1104/pp.101.3.1119 8310052 PMC158736

[B71] Yang Z. Wang X. Gu S. Hu Z. Xu H. Xu C. (2008). Comparative study of SBP-box gene family in Arabidopsis and rice. Gene 407, 1–11. 10.1016/j.gene.2007.02.034 17629421

[B72] Yuan J. S. Reed A. Chen F. Stewart C. N. (2006). Statistical analysis of real-time PCR data. BMC Bioinformatics 7:85. 10.1186/1471-2105-7-85 16504059 PMC1395339

[B73] Zamboni A. Minoia L. Ferrarini A. Tornielli G. B. Zago E. Delledonne M.. (2008). Molecular analysis of post-harvest withering in grape by AFLP transcriptional profiling. J. Exp. Bot. 59, 4145–4159. 10.1093/jxb/ern256 19010774 PMC2639028

[B74] Zheng Z. Qamar S. A. Chen Z. Mengiste T. (2006). Arabidopsis WRKY33 transcription factor is required for resistance to necrotrophic fungal pathogens. Plant J. 48, 592–605. 10.1111/j.1365-313X.2006.02901.x 17059405

