# Supplementary material for: A comprehensive survey of the grapevine VQ gene family and its transcriptional correlation with WRKY proteins
Source: Front Plant Sci. 2015 Jun 12;6:417. doi: 10.3389/fpls.2015.00417 (PMC4464145; doi:10.3389/fpls.2015.00417)
Supplement: Table S1 — List of primers used to quantify VvVQ genes expression by qRT-PCR. [file Table1.DOC]

**Table S1** List of Real Time RT-PCR primers for selected *VvVQ* genes

| **Gene** | **Forward Primer (5’-3’)** | **Reverse Primer (5’-3’)** |
| --- | --- | --- |
| *VvVQ1* | CGGTCAATGGCACAAGTATCACAAG | CCAGCGGAGAAACAATCAACACTAAG |
| *VvVQ2* | AGGACTCCCAGAATATTGAGATGAAG | GACACAGGAGAAGACTGACCAAC |
| *VvVQ3* | ATTCCTAATCCGCATTACCCATTTCC | CTGGAGAAGGTGGAGGTTGAGG |
| *VvVQ4* | CTCCCGTCACCGATGAGTACAC | GACACCATCACAGAACCCAAGTAAG |
| *VvVQ5* | CGGTTGCTAGGTTGCTTGCC | AATATCACATGACTTTCCACGACTCC |
| *VvVQ6* | GGGTGTGGGGAAGAAGAAGATGG | AGCAACGGAAGCCTGAAATTAAGC |
| *VvVQ7*  *VvVQ8*  *VvVQ9*  *VvVQ10*  *VvVQ11*  *VvVQ12*  *VvVQ13* | GTCGGTGGCAATCAAAAGGTTCC  GGTGGCGAGATGATGATGGTGTC  TCCTGGACATGACCGCATTAGAC  GGGGTCTGTGGGGATGATG  TGAAGACATTGATCCACCATGAATCG  GGTGTAACTCCAACCCTGAAAGC  CTCTAGGGGTGAAGGTACAGTGG | ACATCATCCAACTGCTCCATCGG  GGGTGAATGGAGAGGAAACAGAGG  GAGCATAACCATTACCACACAGAGAG  CTGTATGTAAACTATAAACGATGTGC  CAGCAAACCCACCAAAGAACCC  TGAAGCAAAGCAACATCCGAAATG  GACGAGGGATGATGAATGTTGAAGG |
| *VvVQ14*  *VvVQ15* | CTGTATATTTGAGTGGCTGTTGGAG  CATGGCAACAAGAATTACATAGAAGG | GCATAGGCATAATTGGCAATCTGG  GCAGTTGGAGAAGGATTGTGAGG |
| *VvVQ16* | GCTTCTACCTCCACCCTTCAC | CATCCAACAACTCTCAATTACAGTCC |
| *VvVQ17* | TCCATACATTTCTCAATCACACATCC | GAATCGGTCAAACCCAAGTAAAGC |
| *VvVQ18* | CCGTTGGGGTTCGCCTTCTC | GGACTATTGGGATGGTGGACAGC |
| *Actin* | TACAATTCCATCATGAAGTGTGATG | TTAGAAGCACTTCCTGTGAACAATG |
