# Supplementary material for: A comprehensive survey of the grapevine VQ gene family and its transcriptional correlation with WRKY proteins
Source: Front Plant Sci. 2015 Jun 12;6:417. doi: 10.3389/fpls.2015.00417 (PMC4464145; doi:10.3389/fpls.2015.00417)
Supplement: Table S2 — Syntenic blocks of VQ genes between grapevine and Arabidopsis genomes. [file Table2.DOCX]

| **Table S2 Syntenic blocks of *VQ* genes between grape and *Arabidopsis* genomes**   \| **ID** \| **region 1 (Ath)** \| \| \| **region 2 (Grape)** \| \| \| **Gene in the synteny region** \| \| \| \| \| --- \| --- \| --- \| --- \| --- \| --- \| --- \| --- \| --- \| --- \| --- \| \|  \| **Chr** \| **Start** \| **Stop** \| **Chr** \| **Start** \| **Stop** \| **gene 1** \| **gene 1 name** \| **gene 2** \| **gene2 name** \| \| 130 \| Chr1 \| 7404383 \| 7485666 \| Chr18 \| 13811801 \| 16187178 \| AT1G21326 \| *AtVQ3* \| GSVIVT01001661001 \| *VvVQ15* \| \| 330 \| Chr2 \| 17509069 \| 17596629 \| Chr18 \| 20551190 \| 19996866 \| AT2G42140 \| *AtVQ17* \| GSVIVT01033500001 \| *VvVQ6* \| \| 244 \| Chr2 \| 18301350 \| 18339214 \| Chr13 \| 24081260 \| 23858916 \| AT2G44340 \| *AtVQ18* \| GSVIVT01001356001 \| *VvVQ11* \| \| 468 \| Chr3 \| 21438084 \| 21635106 \| Chr18 \| 20551190 \| 19996866 \| AT3G58000 \| *AtVQ25* \| GSVIVT01033500001 \| *VvVQ6* \| \| 731 \| Chr5 \| 18950917 \| 18990772 \| Chr2 \| 7574498 \| 7360572 \| AT5G46780 \| *AtVQ32* \| GSVIVT01013160001 \| *VvVQ3* \| |
| --- | --- | --- | --- | --- | --- | --- | --- | --- | --- | --- | --- | --- | --- | --- | --- | --- | --- | --- | --- | --- | --- | --- | --- | --- | --- | --- | --- | --- | --- | --- | --- | --- | --- | --- | --- | --- | --- | --- | --- | --- | --- | --- | --- | --- | --- | --- | --- | --- | --- | --- | --- | --- | --- | --- | --- | --- | --- | --- | --- | --- | --- | --- | --- | --- | --- | --- | --- | --- | --- | --- | --- | --- | --- | --- | --- | --- | --- |
