# Supplementary figures and images for: A comprehensive survey of the grapevine VQ gene family and its transcriptional correlation with WRKY proteins
Source: Front Plant Sci. 2015 Jun 12;6:417. doi: 10.3389/fpls.2015.00417 (PMC4464145; doi:10.3389/fpls.2015.00417)

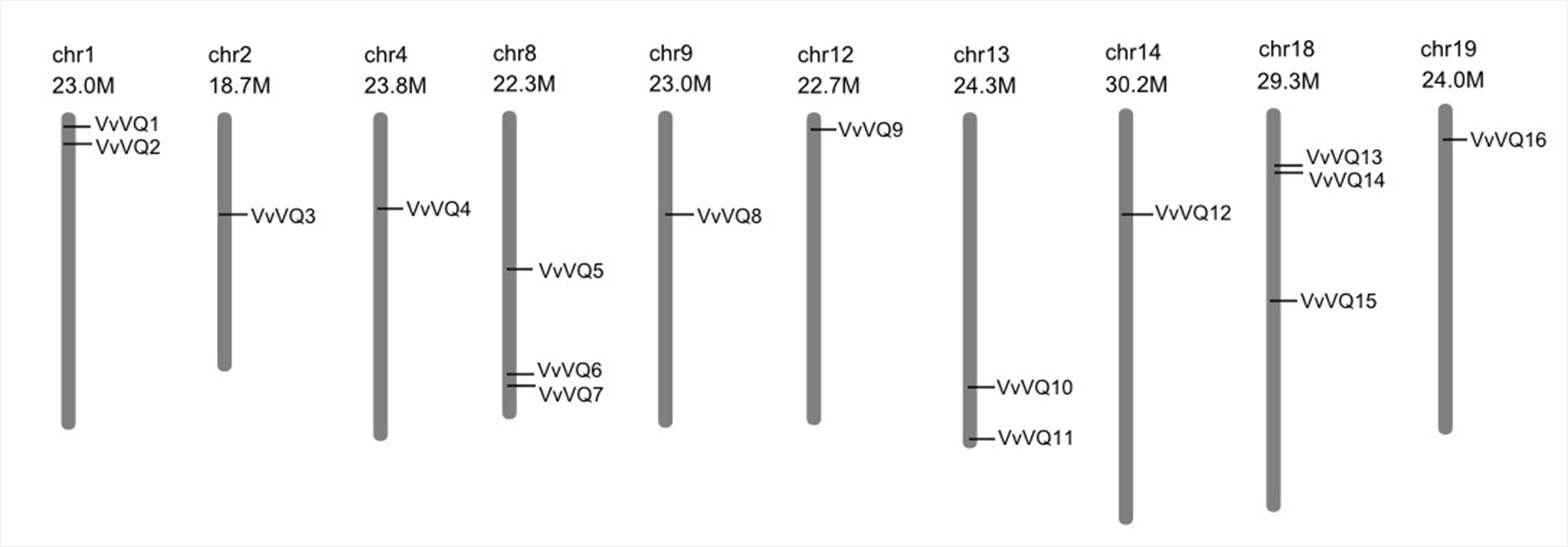

Supplement: Figure S1 — Chromosomal location of VQ genes in grapevine. The chromosomal location of grapevine VQ genes was determined based on the 12X V1 release of the PN40024 grapevine genome (http://genomes.cribi.unipd.it/). The distribution of VvVQ genes was drawn using MapInspect software. [file Image1.TIF]

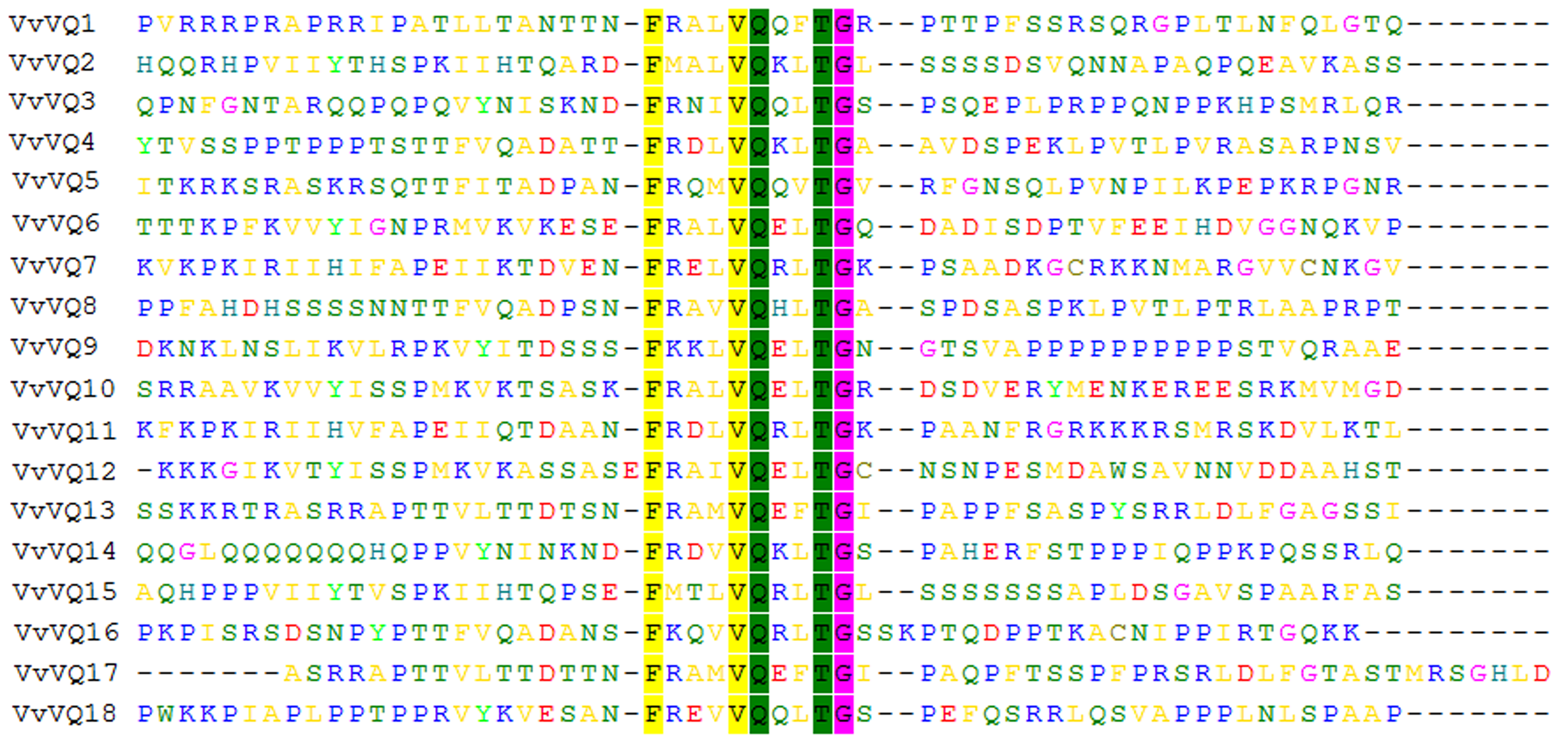

Supplement: Figure S2 — Alignment of VQ domain amino acid sequences of grapevine VQ proteins. Multiple alignment of grapevine VQ domain amino acid sequences was performed using Muscle with default options. [file Image2.TIF]

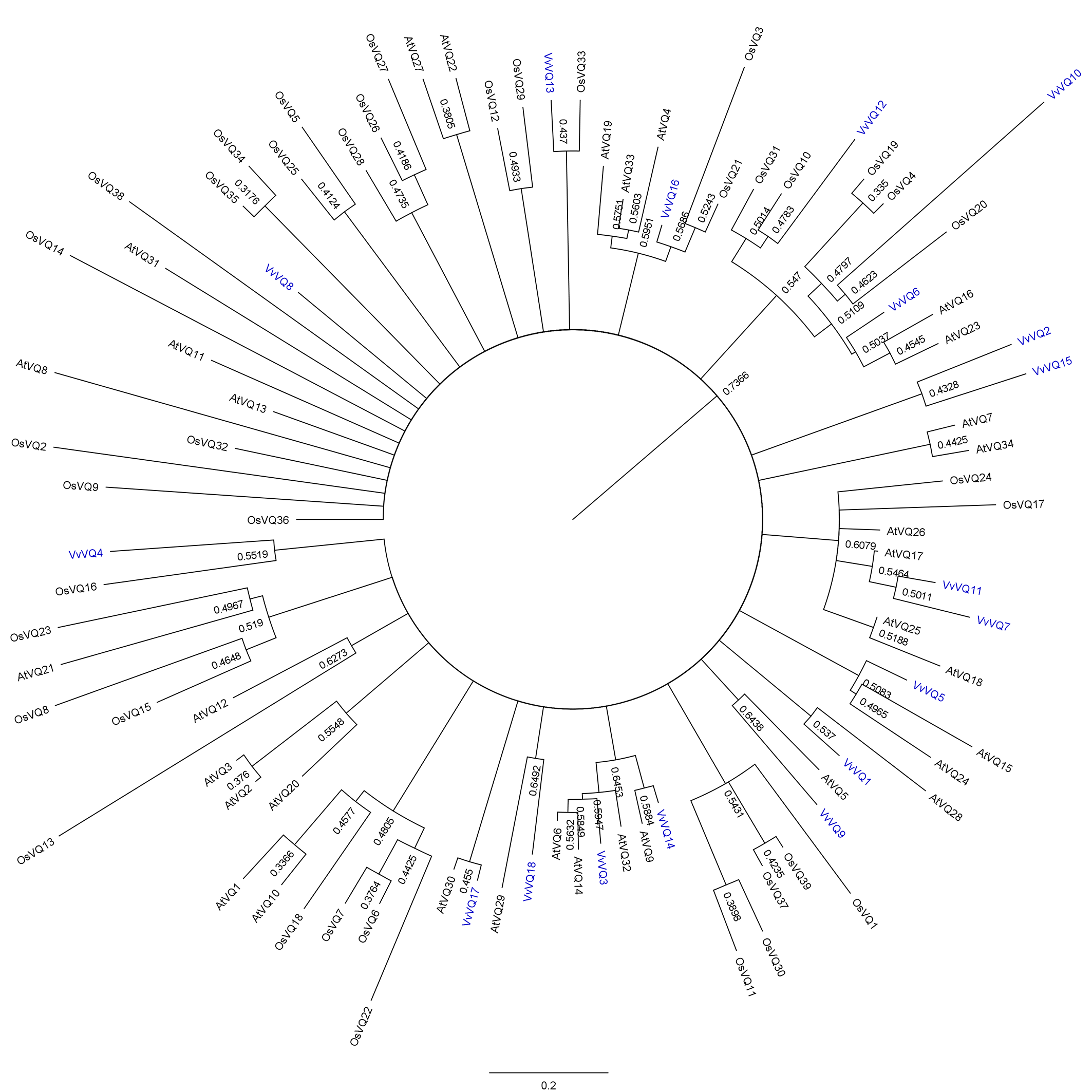

Supplement: Figure S3 — Phylogenetic tree of grapevine, Arabidopsis and rice VQ proteins obtained using a Bayesian-base method. [file Image3.TIF]

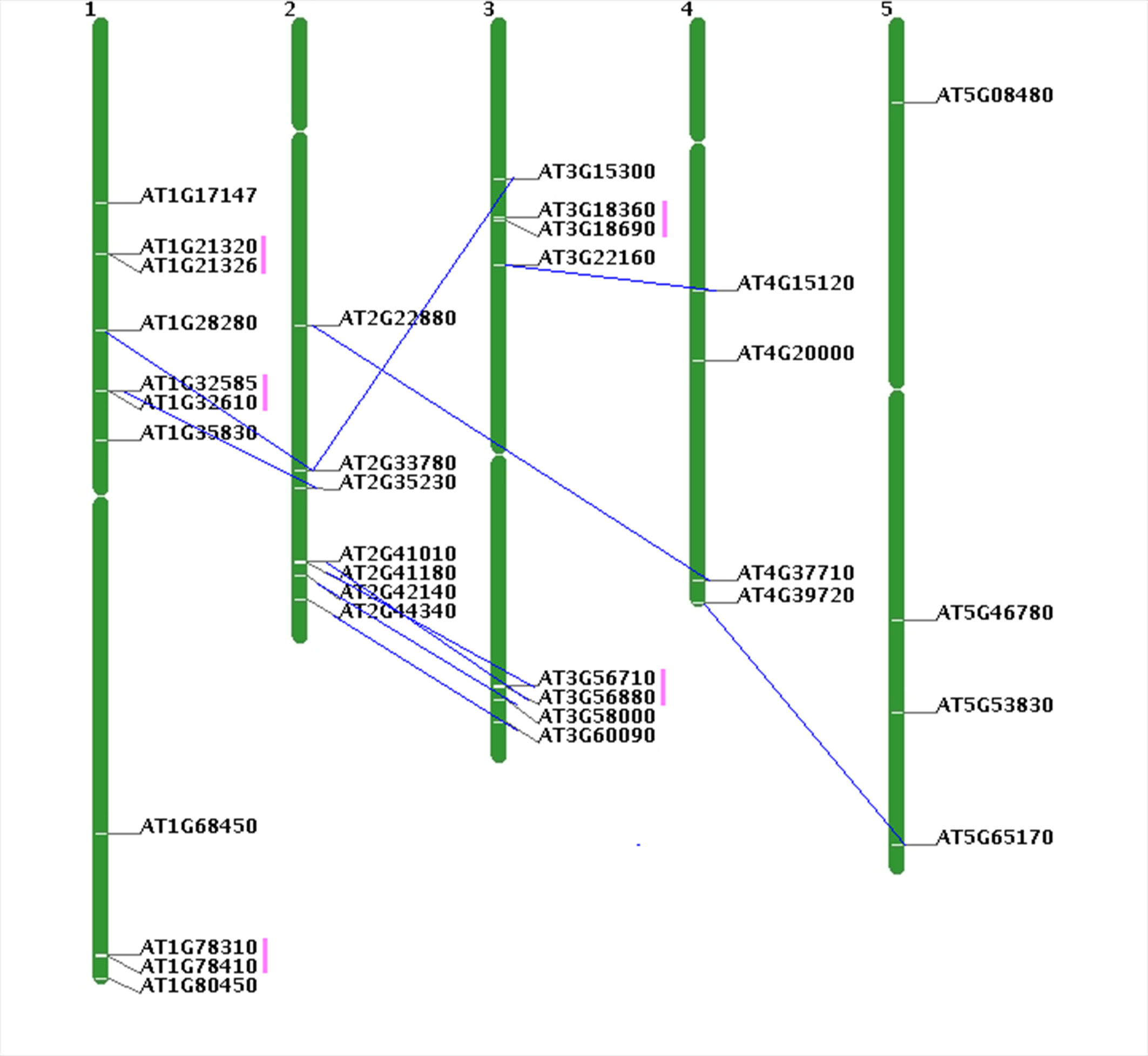

Supplement: Figure S4 — Chromosomal location of VQ genes in Arabidopsis indicating tandem and segmental duplications. Diagram of the five chromosomes of Arabidopsis depicting the location and distribution of 34VQ genes. The segmental duplication genes were connected with a blue line. Tandem duplication genes were indicated with a pink vertical line. [file Image4.TIF]

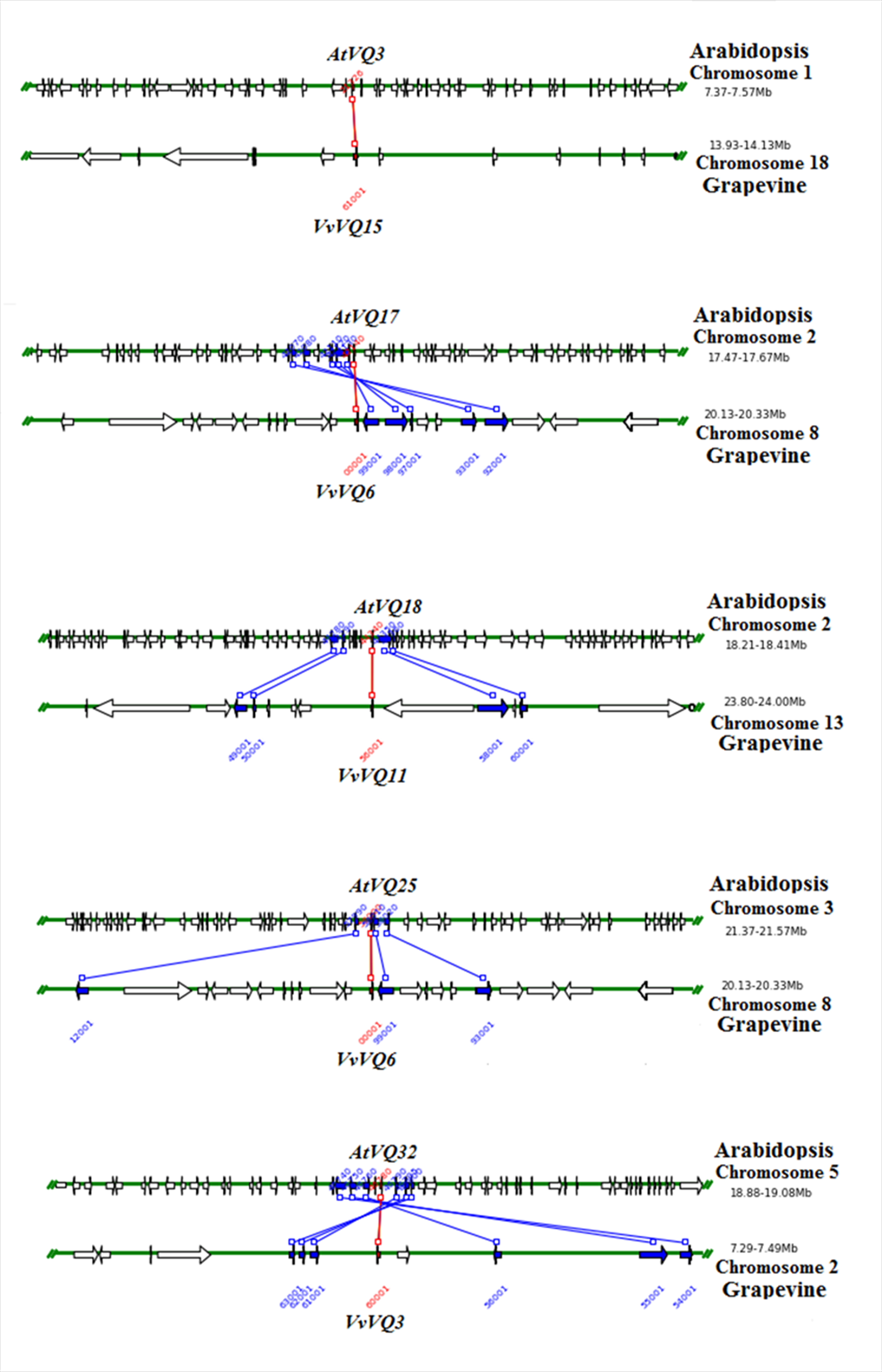

Supplement: Figure S5 — Syntenic blocks of VQ genes (indicated in red) between the grapevine and Arabidopsis genomes. Arrows represent gene loci in the grapevine and Arabidopsis chromosomes. Blue arrows represent duplicated loci in a syntenic block. The data were obtained from the Plant Genome Duplication Database, and syntenic blocks containing grapevine VQ genes were illustrated. [file Image5.TIF]

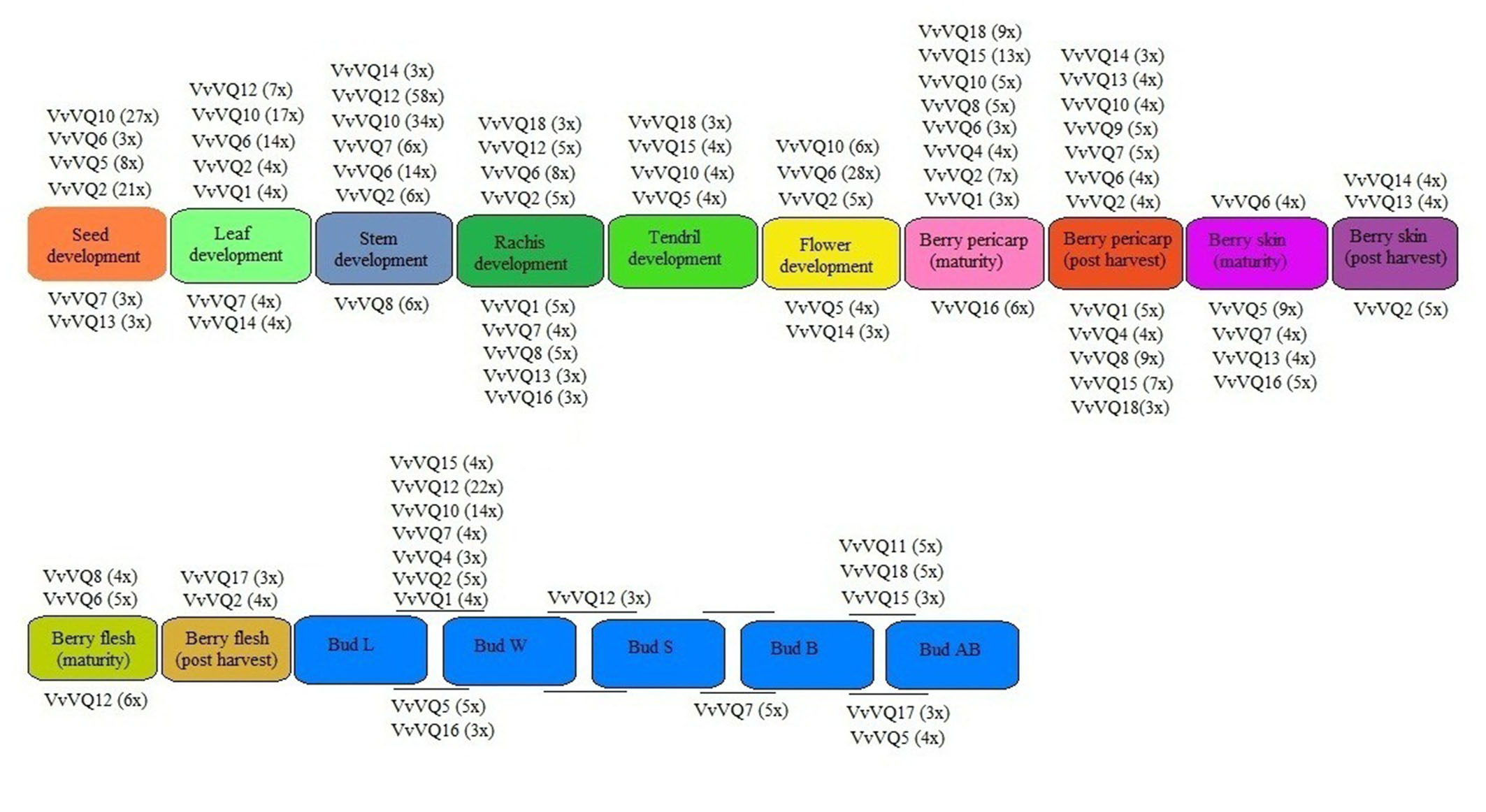

Supplement: Figure S6 — Differential expression of VvVQ genes in various tissues and at different developmental stages based on microarray analysis. The expression levels of the first development stage of each tissue were set at a value of “1” to determine up and down regulation at subsequent stages of development. Tissues containing only one phase were not considered. The genes above and below the colored tissue bar indicated the up-regulated and down-regulated genes, respectively, at this developmental stage. Genes were considered to be differentially expressed if the fold change (up- or down- regulated) was greater than two. [file Image6.TIF]
